# Nascent chains derived from a foldable protein sequence interact with specific ribosomal surface sites near the exit tunnel

**DOI:** 10.1038/s41598-024-61274-1

**Published:** 2024-05-29

**Authors:** Meranda M. Masse, Valeria Guzman-Luna, Angela E. Varela, Ummay Mahfuza Shapla, Rachel B. Hutchinson, Aniruddha Srivastava, Wanting Wei, Andrew M. Fuchs, Silvia Cavagnero

**Affiliations:** 1 Department of Chemistry, University of Wisconsin-Madison, Madison, WI 53706 USA; 2 Present Address: School of Veterinary Medicine, University of Wisconsin-Madison, Madison, WI 53706 USA; 3 Present Address: Department of Food Science, University of Wisconsin-Madison, Madison, WI 53706 USA; 4 Present Address: McGaw Medical Center, Northwestern University, Chicago, IL 60611 USA; 5 Present Address: AIDS Vaccine Research Laboratory, University of Wisconsin-Madison, Madison, WI 53711 USA

**Keywords:** Biopolymers in vivo, Protein folding, Biochemistry, Biophysics, Chemical biology

## Abstract

In order to become bioactive, proteins must be translated and protected from aggregation during biosynthesis. The ribosome and molecular chaperones play a key role in this process. Ribosome-bound nascent chains (RNCs) of intrinsically disordered proteins and RNCs bearing a signal/arrest sequence are known to interact with ribosomal proteins. However, in the case of RNCs bearing foldable protein sequences, not much information is available on these interactions. Here, via a combination of chemical crosslinking and time-resolved fluorescence-anisotropy, we find that nascent chains of the foldable globin apoHmp_1–140_ interact with ribosomal protein L23 and have a freely-tumbling non-interacting N-terminal compact region comprising 63–94 residues. Longer RNCs (apoHmp_1–189_) also interact with an additional yet unidentified ribosomal protein, as well as with chaperones. Surprisingly, the apparent strength of RNC/r-protein interactions does not depend on nascent-chain sequence. Overall, foldable nascent chains establish and expand interactions with selected ribosomal proteins and chaperones, as they get longer. These data are significant because they reveal the interplay between independent conformational sampling and nascent-protein interactions with the ribosomal surface.

## Introduction

Recent evidence suggests that the ribosome plays an active role in cotranslational protein folding and solubility^1–8^. During translation, the nascent chain traverses the ribosomal exit tunnel, which is ca. 80–100 Å long, 10–35 Å wide^9–12^ and typically fits 30–40 nascent residues^13–18^. Within the ribosomal exit tunnel and its nearby regions across the highly negatively charged outer surface of the ribosome^19^, nascent chains encoding single-domain proteins become compact^20–22^ and acquire some secondary^18,23–26^ and tertiary structure^5,27–31^. This set of observations proves the importance of the ribosome in nascent-protein structure formation.

During translation, the ribosome influences nascent protein chains at different levels. First, it renders nascent chains soluble relative to the corresponding ribosome-released proteins, thereby supporting cotranslational events devoid of undesirable aggregation^7^. Second, the inner geometry of the ribosomal exit tunnel favors formation of secondary nascent-chain structure, especially of α-helical nature^18,26,32–34^. In addition, the ribosomal exit tunnel and vestibule enable acceleration of folding – but not unfolding – of a small single-domain protein, thereby stabilizing nascent protein chains relative to their free state in solution^35^. This effect was ascribed primarily to electrostatic interactions between nascent proteins and ribosome^35^. On the other hand, the ribosome may also destabilize single-protein domains, in case the domain is far removed from the peptidyl transferase center^36^. Other studies support either RNC stabilization or destabilization by the ribosome^5,31,37^. Collectively, these results highlight the influence of the ribosome on nascent protein folding.

The ribosome is also known to establish physical noncovalent interactions with some nascent chains. As summarized in Table 1, these interactions were identified in a variety of experimental studies and can be divided into three categories. Namely, *(i)* interactions between the ribosome and nascent chains carrying an N-terminal signal sequence^18,38–42^, *(ii)* interactions between the ribosome and nascent chains bearing a C-terminal ribosome-stalling or arrest sequence^38,43–48^, and *(iii)* interactions between the ribosome and nascent chains bearing no N- or C-terminal tags^26,49,50^. Additional studies are consistent with the presence of ribosome-nascent-chain interactions, though they do not directly prove their existence^51–54^.

**Table 1:** Summary of known interactions between RNCs and the ribosome, based on cryo-EM and chemical crosslinking. Studies with nascent peptides (as opposed to proteins, e.g., see Wilson and Beckmann^1^) were omitted, as this compilation focuses on proteins.

RNC	Technique	Interacting ribosomal protein
**Proteins with an N-terminal signal sequence**		
Leader peptidase^40^	Photo-crosslinking	L4, L22 and L23
Two regulatory ribosome stalling peptides^38^	cryo-EM	L4 and L17
Transmembrane segment of 111p membrane protein^18^	Photo-crosslinking	Not determined
Signal anchor of FtsQ protein^42^	Photo-crosslinking	L23 and L29
pOmt^39^	Photo-crosslinking	L23 and L24
EsP1-25^41^	Photo-crosslinking	L23 and L24
**Proteins with a C-terminal ribosome-stalling sequence**		
TnaC^43^	Photo-crosslinking	L22 and L24
TnaC^44^	cryo-EM	L22
SecM^38^	cryo-EM	L22 and L23
SecM^45^	Mutagenesis	L22
SecM^46^	cryo-EM	L23
Signal anchor of Dap2 Protein^47^	Photo-crosslinking	RpI4, Rp17 and RpI29
SecM^48^	NMR	Not determined
SecM, with a ribosome bearing an extended L23 loop^108^	cryo-EM	L23 and L24
**Proteins with no N- or C-terminal tag**		
Phosphorylated insulin receptor (PIR) (intrinsically disordered protein)^49^	Chemical crosslinking	L23
Cold shock protein A (CspA)^26^	cryo-EM	L4, L22 and L23
Apomyoglobin (apoMb)^50^	cryo-EM	L4, L22 and L23

In summary, ribosome-bound nascent chains (RNCs) lacking or including signal or arrest sequences are known to interact with ribosomal proteins. However, only very few RNCs bearing foldable protein sequences and lacking linkers or signal/arrest tags have been explicitly characterized to date, in terms of experimentally detectable interactions with the ribosome.

Further, little is known about how the nascent chain may affect certain components of the ribosome. For instance, empty 70S ribosomes are known to be more prone to dissociation than ribosomes bearing both mRNA and peptidyl tRNA. This conclusion was reached upon addition of either ribosome-dissociation factors^55^ or Hofmeister cosolutes^56–58^. Other researchers established a similar finding upon depletion of magnesium ions^59–61^. In a different study, addition of Hofmeister salts were employed to show that translation initiation complexes (including 70S in complex with initiator tRNA) disassemble more easily than peptidyl tRNAs bearing nascent chains^62^. Ribosomes carrying longer nascent chains were found to be less prone to dissociation^62^. Other studies examined the effect of magnesium ions and other Hofmeister ions on empty-70S-ribosome disassembly and how it changes sedimentation coefficients^63^. Yet, there is only a limited number of studies targeting the effect of non-Hofmeister denaturing agents on the ribosome. For instance, it is known that the 30S subunit disassembles in the presence of 6 M urea^64^. In addition, urea lowers the melting temperature and sedimentation coefficient of the 50S ribosomal subunit^65^. The 30S subunit is more sensitive to thermal denaturation than the 50S subunit, and the 70S ribosome is most thermally stable^65^. In addition, 70S ribosomes bearing a nascent chain are less prone to chemical denaturation than empty ribosomes^36^.

Here, we address the lack of knowledge on nascent-chain/ribosome interactions by exploring them in the case of ribosome-bound nascent chains (RNCs) of increasing length belonging to a foldable protein sequence. We find that RNCs up to chain length 140 interact only with one ribosomal protein (r-protein), i.e., L23, in the vicinity of the ribosomal exit tunnel. This result is surprising because the ribosomal surface near the tunnel exit bears several r-proteins. A wider interaction network, including one additional ribosomal protein and the trigger factor (TF) chaperone, gets established as the nascent chain elongates up to 189 residues. The populations of RNC-interacting proteins evolve as a function of chain elongation. Specifically, interactions with r-proteins get partially or completely replaced by interactions with the TF molecular chaperone, as TF concentration increases up to physiologically relevant values. In order to gain additional insights on the potential stabilizing role of the interactions, we also investigate the effect of the above RNC/r-protein interactions on the bacterial ribosome. The apparent stability of the complexes between RNCs of foldable and intrinsically disordered protein sequences and specific r-proteins is weak and, surprisingly, does not vary significantly with RNC sequence, length, net charge and hydrophobicity. Hence, we propose that the ribosome provides unbiased thermodynamic assistance to nascent chains regardless of their electrostatic and nonpolar character. As an ancillary finding, we also show that the apparent thermodynamic stability of the peptidyl transferase center (PTC) and all ribosomal proteins is not affected by RNC-ribosome interactions. Further, short peptidyl-tRNAs (snc-tRNAs) stabilize the 70S ribosome against denaturation by the non-Hofmeister cosolute urea, suggesting a multi-step model for the disassembly of ribosome-RNC complexes.

In all, our results highlight the supporting role of the ribosome for newly synthesized protein chains, showing that it establishes interactions with RNCs via specific r-proteins.

## Results and discussion

### Experimental design

**Figure 1:**
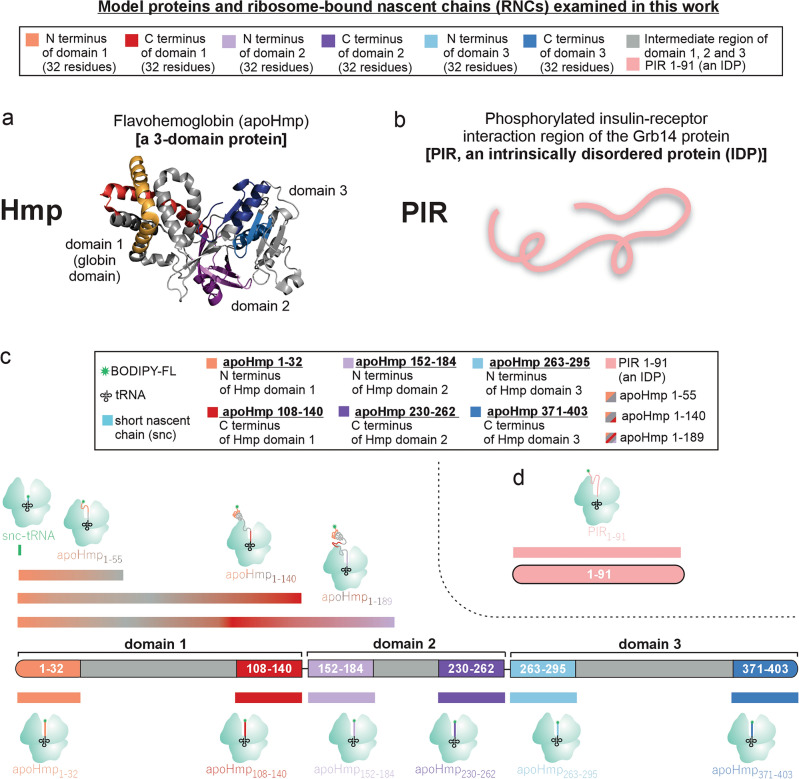
Cartoons illustrating model proteins and constructs employed in this work. (**a**) Structure of *E. coli* flavohemoglobin (Hmp), the model foldable protein used in this study. Hmp has three domains (shown in red, purple and blue). PDB code: 1GVH. (**b**) Cartoon illustrating the phosphorylated insulin receptor interacting region of the Grb14 protein from rat (PIR). PIR is the model intrinsically disordered protein used in this work. (**c**) Schematic representation of the length and sequence location of the three domains of Hmp. The specific RNC constructs of the apo form of Hmp used in this work (denoted here as apoHmp) are also shown, with the respective chain lengths listed as subscripts. (**d**) Linear bar illustrating the 91-residues length of PIR and its respective RNC.

This work focuses on ribosome-bound nascent chains (RNCs) derived from *Escherichia coli* flavohemoglobin (Hmp, Fig. 1a) and from the phosphorylated insulin receptor interacting region (PIR) of the growth factor receptor-bound protein 14 from *Rattus norvegicus* (Fig. 1b)*.* The Hmp protein comprises three domains, an N-terminal heme-binding (domain 1), a flavin adenine dinucleotide-binding (domain 2) and a C-terminal nicotinamide adenine dinucleotide-binding domain (domain 3), as shown in Fig. 1c^66^. Several RNC chain lengths were examined, and all pertinent constructs are shown as solid bars in Fig. 1c. Hmp plays a key role in O_2_, NO and CO transport in *E. coli*, and is involved in a variety of signaling pathways^67,68^. Importantly, previous studies established that the N-terminal globin domain of Hmp is stable and folded even in its cofactor-free apo form, known as apoHmpH^69^. Our second target protein, PIR, is intrinsically disordered^70^, i.e., an IDP (Fig. 1b). The specific nascent-chain constructs of both proteins analyzed in this work are schematically illustrated in Fig. 1c,d.

**Figure 2:**
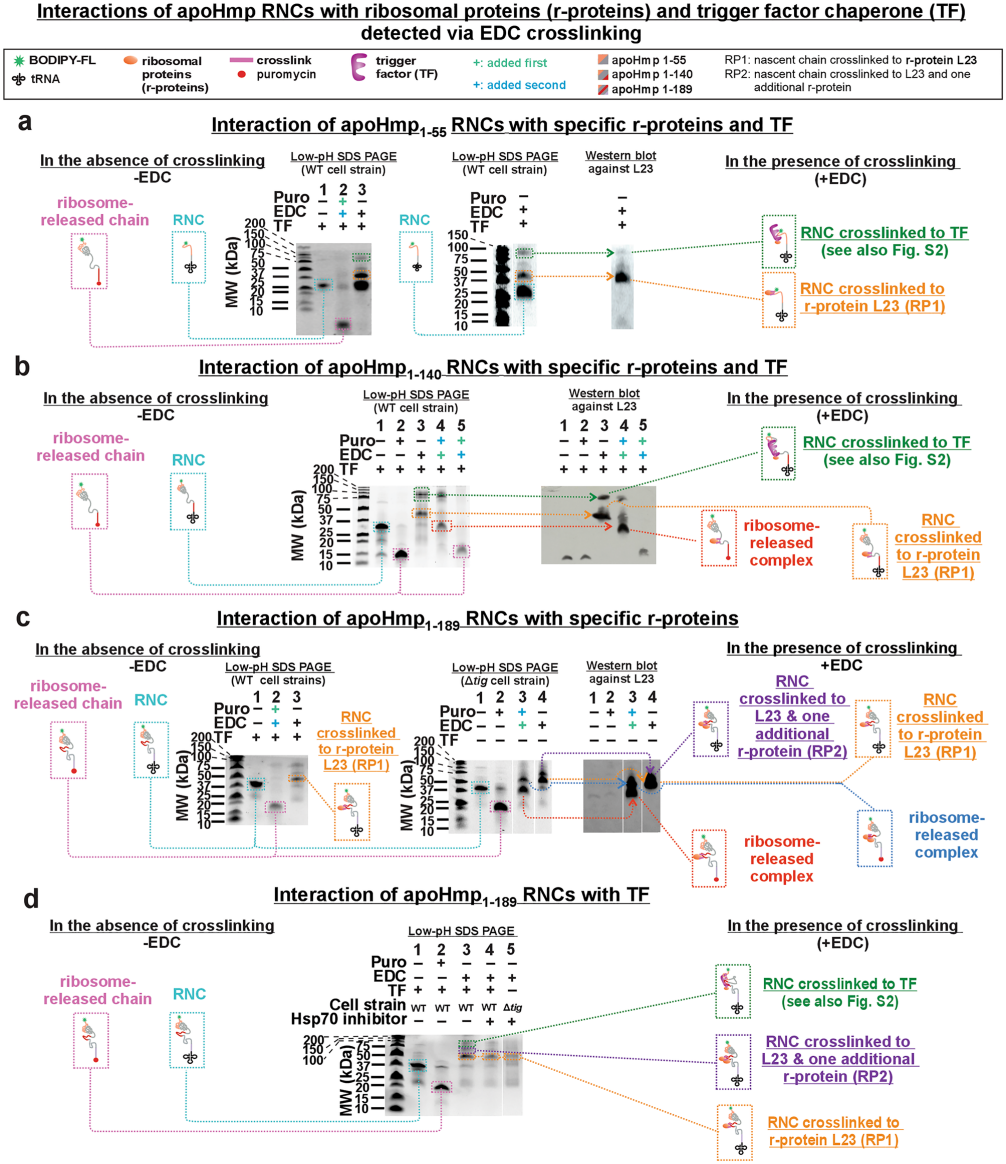
Crosslinking patterns of apoHmp_1–140_ and apoHmp_1–189_ RNCs and identification of interacting ribosomal proteins. (**a**) SDS-PAGE and Western blot data identifying r-proteins interacting with apoHmp_1–55_ RNCs in the presence of the EDC crosslinker and the ribosome-release agent puromycin. Data show that the L23 r-protein interacts with apoHmp_1–55_ RNCs. Corresponding data employing antibodies against L24 and L29 r-proteins, showing no interactions, are available in the SI. Here, RNC’s are denoted as nascent chains bound to tRNA, in which the ribosome has been removed due to SDS and heat from gel analysis. (**b**) SDS-PAGE and Western blot data identifying r-proteins interacting with apoHmp_1–140_ RNCs in the absence and presence of the EDC crosslinker and the ribosome-release agent puromycin. Data show that the L23 r-protein interacts with apoHmp_1–140_ RNCs. Corresponding data employing antibodies against L24 and L29 r-proteins, showing no interactions, are available in the Supplementary Information. (**c**) Side-by-side SDS-PAGE and Western blot data identifying the interaction network of apoHmp_1–189_ RNCs in the absence and presence of EDC, puromycin and TF chaperone. The L23 r-protein is found to interact with apoHmp_1–189_ RNCs. (**d**) Low-pH SDS-PAGE analysis of apoHmp_1–189_ RNCs in the absence and presence of the EDC crosslinker, TF chaperone and the RNC ribosome-release agent puromycin. Corresponding data employing antibodies against L24 and L29 r-proteins, showing no interactions, are available in the Supplementary Information. Uncropped gel images for all panels are shown in Supporting Figures S10 and S11. Pertinent *E. coli* cell strains are listed above the SDS-PAGE gel. Note that the wild-type (WT) cell-free system, corresponding to the WT strain, contains the following chaperone concentrations: TF (2–15 nM), DnaK (0.5 µM), DnaJ (0.04 µM) and GrpE (0.05 µM). In addition, note that lanes 4 and 5 include the KLR-70 Hsp70 inhibitor, added at 0.2 mM concentration.

RNCs were produced via an in-house-made *E. coli* cell-free system^20,71^, at a final concentration of ca. 30 nM, which was assessed as described^72^. The ribosome-bound identity of all nascent chains was verified with + /- puromycin assays^20,71^ (see Figs. 2, 6–8, S1, S8, S10–S13). The chain-length homogeneity of RNCs was verified by assessing the predominance of a single band at the expected molecular weight (tRNA: 26–28 kDa, plus nascent-chain pertinent mass) via low-pH SDS-PAGE^73^. Ribosome-nascent-protein interactions and their urea sensitivity were probed with via the well-characterized zero-length chemical crosslinker 1-ethyl-3-[3-dimethylaminopropyl] carbodiimide hydrochloride (EDC)^49,74,75^. RNC crosslinking was verified by low-pH SDS-PAGE^73^ and crosslinked r-protein identity was assessed by Western blotting in the absence and presence of the trigger factor (TF) chaperone. Notably, EDC leads to unequivocal identification of RNC/r-protein interactions^50^, yet it tends to underestimate interacting fractions^49^. Nonetheless, in the presence of appropriate controls, it is an extremely valuable tool to detect the existence of protein–protein interactions within the ribosome-nascent-chain complex. In addition, relative changes in the extent of the interactions, for any given ribosome-nascent-chain complex (RNC) as a function of environmental changes (e.g., variable urea or chaperone concentrations) were also qualitatively assessed. Site-specific fluorescence labeling of nascent proteins at their N terminus enables focusing exclusively on interactions involving the nascent protein. Low-pH-gel and Western-blot were collected to explore interactions between nascent chains and r-proteins. It is worth noting that EDC does not have high accessibility within the exit-tunnel core^49^. Therefore, detection of interactions within the tunnel core is not expected, within our experimental setup.

It is also important to mention that, under our experimental conditions, EDC does not report on interactions involving nascent protein chains and ribosomal RNA (rRNA). In the presence of imidazole, crosslinks between RNA 5' phosphate and aliphatic amines of proteins are known to take place^74^. However, our samples did not contain imidazole, and this chemical would anyways be unable to detect interactions not involving the 5’ end of RNA. Therefore, even in the presence of imidazole, EDC would likely underestimate all potential interactions with RNA. Thus, interactions between nascent proteins and rRNA are beyond the scope of this study.

The compaction, tumbling rates, size (expressed in terms of approximate number of residues) and local-motion amplitude of non-interacting RNC regions were assessed via fluorescence depolarization decays in the frequency domain. In this way, it was possible to gain complementary and more comprehensive insights into RNC conformational characteristics. The apparent thermodynamic stability of r-proteins was collectively assessed by Trp fluorescence emission spectroscopy as a function of urea concentration. The apparent stability of the peptidyl transferase center (PTC) of the ribosome in the presence of a variety of RNCs was evaluated by urea titrations upon detection via a puromycin-release assay. Finally, the empty-ribosome and RNC assembly status of the ribosome, in terms of 30S, 50S and 70S subunits, was assessed via sucrose-gradients and negative-staining transmission electron microscopy.

### ApoHmp RNCs of increasing length interact with ribosomal protein L23

We elected to probe whether apoHmp RNCs interact with the L23, L24 and L29 r-proteins, which reside within the vestibule of the ribosomal exit tunnel and the adjacent outer surface of the ribosome. We explored the interaction patterns of three representative nascent chains, namely apoHmp_1–55_, apoHmp_1–140_ and apoHmp_1–189_. The data for these RNCs are shown in Fig. 2. A side-by-side comparison between low-pH SDS-PAGE^73^ and Western blots indicate that all three nascent proteins interact with ribosomal protein L23. Western blotting carried out with antibodies against ribosomal proteins L24 and L29, shown in the Supplementary Information (Fig. S1), indicates no evidence for interactions between the L24 and L29 r-proteins and the target RNCs. The surface-charge distribution of all 50S r-proteins facing the ribosomal outer surface^49^ is similarly rich in negatively charged and solvent-exposed EDC-reactive residues (Lys, Asp, Glu)^74,75^. Therefore, we conclude that the detected interactions with r-proteins facing the outer ribosomal surface are unlikely to include false negatives^49^. Therefore, we conclude that apoHmp RNCs interact exclusively with ribosomal protein L23. In contrast, intrinsically disordered PIR_1–91_ RNCs, analyzed in previous studies^49^, interact with both the L23 and L29 ribosomal proteins.

Under our experimental conditions, the fraction of interacting RNCs is different, for nascent chains derived from apoHmp_1–55_, apoHmp_1–140_, apoHmp_1–189_ (Fig. 2a–c and Table 2) and intrinsically disordered PIR_1–91_^49^. Indeed, apoHmp_1–55_ and PIR_1–91_ crosslink only in part, unlike apoHmp_1–140_ and apoHmp_1–189_ RNCs, which are nearly 100% crosslinked. On the other hand, the larger extent of crosslinking of the foldable apoHmp_1–140_ and apoHmp_1–189_ RNCs relative to apoHmp_1–55_ and PIR may be mainly a consequence of the greater number of EDC-reactive residues (Lys, Asp, Glu)^74,75^ of apoHmp_1–140_ (25 EDC-reactive residues, ca. 20 beyond the tunnel core) and apoHmp_1–189_ (36 EDC-reactive residues, ca. 30 beyond the tunnel core) relative to apoHmp_1–55_ (11 EDC-reactive residues, ca. 5 beyond the tunnel core) and PIR (14 EDC-reactive residues, ca. 12 beyond the tunnel core). In support of this argument (see sections below), the urea sensitivity of the L23 / RNC complexes is similar for all RNCs, suggesting comparable interaction strengths. To provide more quantitative evidence and further illustrate this point, Table 2 illustrates the percent of EDC crosslinking of the 1–55, 1–140 and 1–189 apoHmp RNCs analyzed in this work. While the 1–55 shorter RNC crosslinks to the L23 r-protein to a more moderate degree than the 1–140 and 1–189 constructs, this apparent difference in reactivity vanishes when the number of EDC-reactive residues beyond the tunnel core is taken into account.

**Table 2:** Percent EDC crosslinking of apoHmp RNCs, assessed with the ImageJ software.

RNC	% EDC crosslinking (avg. ± SE)	Normalized % EDC crosslinking^a^ (avg. ± SE)
apoHmp_1–55_	27.4 ± 0.1	5.5 ± 0.1
apoHmp_1–140_	92 ± 3	4.6 ± 0.2
apoHmp_1–189_	85 ± 1	2.8 ± 0.1

^a^per EDC-reactive residue beyond the ribosomal tunnel core.

Importantly, given that fluorescence anisotropy-decay data (see later sections) show that apoHmp_1–140_ and apoHmp_1–189_ RNCs have dynamic and independently tumbling N-terminal compact regions, it is clear that the RNC regions interacting with the ribosomal surface cannot include any significant fraction of N-terminal residues belonging to the compact region.

**Figure 3:**
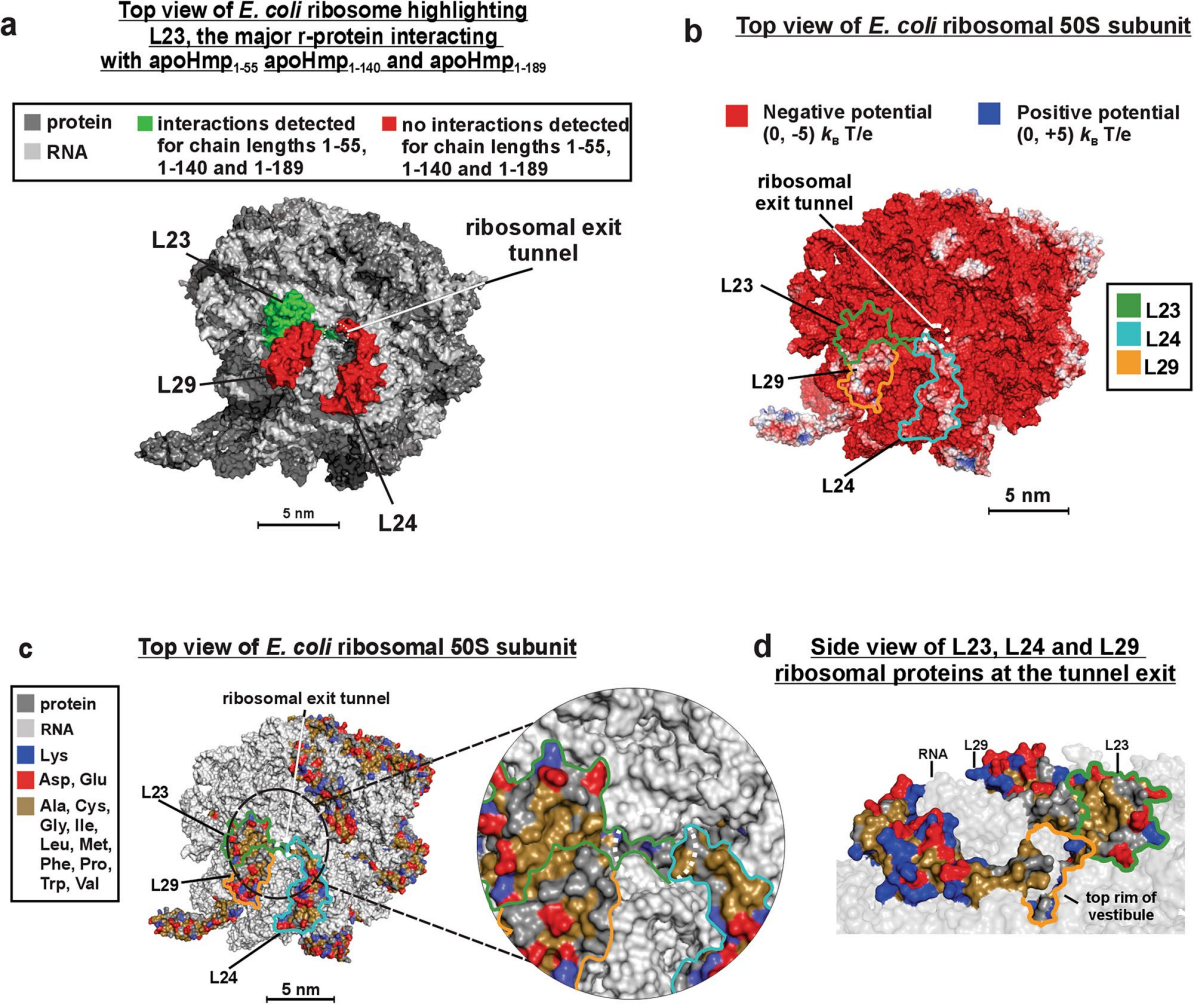
Characteristics of the 70S *E. coli* ribosome. (**a**) Top view of *E. coli* 50S ribosomal subunit highlighting the r-proteins that either interact (green) or do not interact (red) with apoHmp_1–55_, apoHmp_1–140_ and apoHmp_1–189_ RNCs. (**b**) Top view of the 50S subunit of the *E. coli* ribosome displaying the electrostatic surface potential map and highlighting relevant r-proteins. (**c**) Top view of 50S *E. coli* ribosome highlighting the charged and nonpolar residues of r-proteins. (**d**) Side view of r-proteins near the vestibule of the ribosomal exit tunnel. The images in panels b, c and d have been modified from reference (50) under a Creative Commons Attribution 4.0 International license.

In the case of the longest RNCs analyzed in this work, corresponding to apoHmp_1–189_, we found an additional interacting complex of higher molecular weight, which we denote as RP2 (Fig. 2c). The corresponding population includes r-protein L23, according to Western blotting, see Fig. 2c, and one additional unidentified protein of c.a. 6–10 kDa, according to molecular weight arguments. Our Western blots indicate that L29 (7 kDa) is not present in the RP2 band (Fig. S1f.). Yet, other cytoplasmic *E. coli* chaperones and ribosome interactors (GroEL, GroES, SecB, DnaK/DnaJ/GrpE, SRP and ClpB; MW range: 48–80 kDa) are ruled out, as they would appear well above the RP2 complex in our gels (Fig. 2c,d). Due to its close spatial proximity to L23 (Fig. 3a–d) and based on the above-mentioned molecular-weight arguments, it is possible that RP2 comprises both L23 and L29. However, our monoclonal antibodies against r-protein L29 were unable to capture an L29 epitope, as part of the crosslinked complex adsorbed onto the PVDF membrane. To further test for the possibility that L29 being part of the RP2 interacting protein pair, additional experiments in the presence of polyclonal antibodies against L29 will be carried out in the future.

The data in Figs. 2 and S2 also show that a fraction of the apoHmp_1–55_, apoHmp_1–140_ and apoHmp_1–189_ RNCs interacts with the trigger factor (TF) chaperone. The presence of these contacts was assessed upon comparing denaturing gels for data collected with wild-type (WT) and TF-depleted (*Δtig*) *E. coli* cell strains. Indeed, RNC / TF interactions are already known to exist from previous literature, especially for nascent proteins longer than ca. 100–110 residues^8,42,76–79^ Previous studies also showed that TF interacts with client proteins that bear a fairly expanded conformation, in their bound state^80–82^.

On the other hand, the small observed fraction of apoHmp_1–55_ interacting with TF (Fig. 2a) is unexpected. This result implies the presence of a highly stretched conformation of this short (55-residue) RNC, which must reside mostly within the 80–100 Å-long ribosomal exit tunnel. Yet, apoHmp_1–55_ manages to reach out to the TF chaperone, which is known to dock onto the outer surface of the ribosome via the L23 and L29 r-proteins. This conformational stretching experienced by a small fraction of the short apoHmp_1–55_ RNC is fascinating and unprecedented. Indeed, the presence of L23-docked and TF-docked apoHmp_1–55_ nascent-chains suggests that cotranslational conformational sampling can take place even in the case of a fairly short RNC.

TF and L23 are known to interact with one another on the ribosome^77,82,83^, though we presently cannot explicitly discriminate whether the nascent chains interact with L23 and TF, or if the nascent chain interacts with TF, which in turn interacts with L23. Here, we propose the simplest scenario namely that RNCs interact with TF and, in turn, TF interacts with L23, which is known to be the TF docking site on the ribosome^2^.

Finally, in this work we only analyzed the behavior of RNCs in the presence of moderate concentrations of the 70 kDa Hsp70 chaperone. Hsp70 was studied in the context of the DnaK/DnaJ/GrpE chaperone system, denoted here as K/J/E. Now, the wild-type (WT) cell-free system used in Fig. 2a,b,d contains K/J/E at 0.5, 0.04, 0.05 µM concentrations, respectively, which are significantly lower than physiologically relevant values. Interestingly, at these low K/J/E concentrations, none of these chaperones is bound to the apoHmpH_1–55_, apoHmp_1–140_ and apoHmp_1–189_ resuspended RNCs, as shown in Fig. 2a,b,d. Therefore, the Hsp70 chaperone does not bind the RNCs studied in this work. The effect of higher, more physiologically relevant (20–50 µM) K/J/E concentrations^84^ will be studied elsewhere.

In all, our data show that apoHmp_1–55_, apoHmpH_1–140_ and apoHmp_1–189_ RNCs interact with either the L23 r-protein alone (apoHmp_1–55_ and apoHmpH_1–140_, Fig. 3a), with L23 and another ribosomal protein (apoHmpH_1–189_), or with the TF chaperone (all RNCs, including apoHmp_1–55_). We propose that these two classes of interactions (i.e., with r-proteins and with TF) play a similar chaperone-like role. This concept is consistent with previous studies, which showed that the ribosome serves as a nascent-chain solubilizing agent even in the absence of chaperones^7^. The solvent-exposed nonpolar patch of the L23 r-protein, highlighted in Fig. 3c,d, is known to interact with another nascent globin^85^. It is therefore possible that L23 establishes contacts with nonpolar regions of RNCs. Future work will focus on genomic *E. coli* r-protein modifications aimed at disrupting the detected interactions. Interestingly, the fact that both RNCs of the foldable apoHmp and the intrinsically disordered PIR_1–91_^49^ chains interact with L23 shows that the RNC-L23 contacts are unrelated to the independent foldability of nascent chains. Moreover, the fact that PIR_1–91_ RNCs also interact with L29, unlike apoHmp_1–55_ and apoHmp_1–140_ RNCs (which only interact with L23), suggests that the intrinsically disordered nascent chain experiences more extensive “sampling” of the outer ribosomal surface. This observation is particularly interesting considering that PIR_1–91_ carries only 91 residues and interacts with both L23 and L29, while the longer apoHmp_1–140_ chain (140 amino acids), only interacts with L23. This result can be rationalized upon considering that apoHmp_1–140_ RNCs have a compact N-terminal region comprising > 60 residues (see next section), while PIR_1–91_ RNCs lack any N-terminal compaction and are therefore more extended^49^.

### Ribosome-bound apoHmp nascent chains of variable length have a compact N-terminal region

Next, we performed fluorescence depolarization decay experiments in the frequency domain^86–88^ to probe the rotational dynamics of nascent chains encoding foldable sequences. This technique has been previously employed to assess the rotational correlation time (τ_c_) and amplitude of rotational motions of RNCs^7,20,21,51,89^. The goal of this experiment was to determine whether RNCs harboring long nascent chains display any degree of compaction. We focused on RNCs of apoHmp_1–140_, corresponding to the N-terminal domain 1 of Hmp (Fig. 1a), and RNCs of apoHmp_1–189_, which comprise Hmp’s domain 1 and an additional 49 C-terminal residues belonging to domain 2 (Fig. 1c). Nascent proteins were site-specifically labeled at their N terminus with the BODIPY-FL fluorophore as described^20^. Once information on nascent-chain compaction is in hand, the interplay between ribosome and nascent-chain interactions, and their sensitivity to urea denaturation can be more rationally explored and understood, as apparent in the sections below.

**Figure 4:**
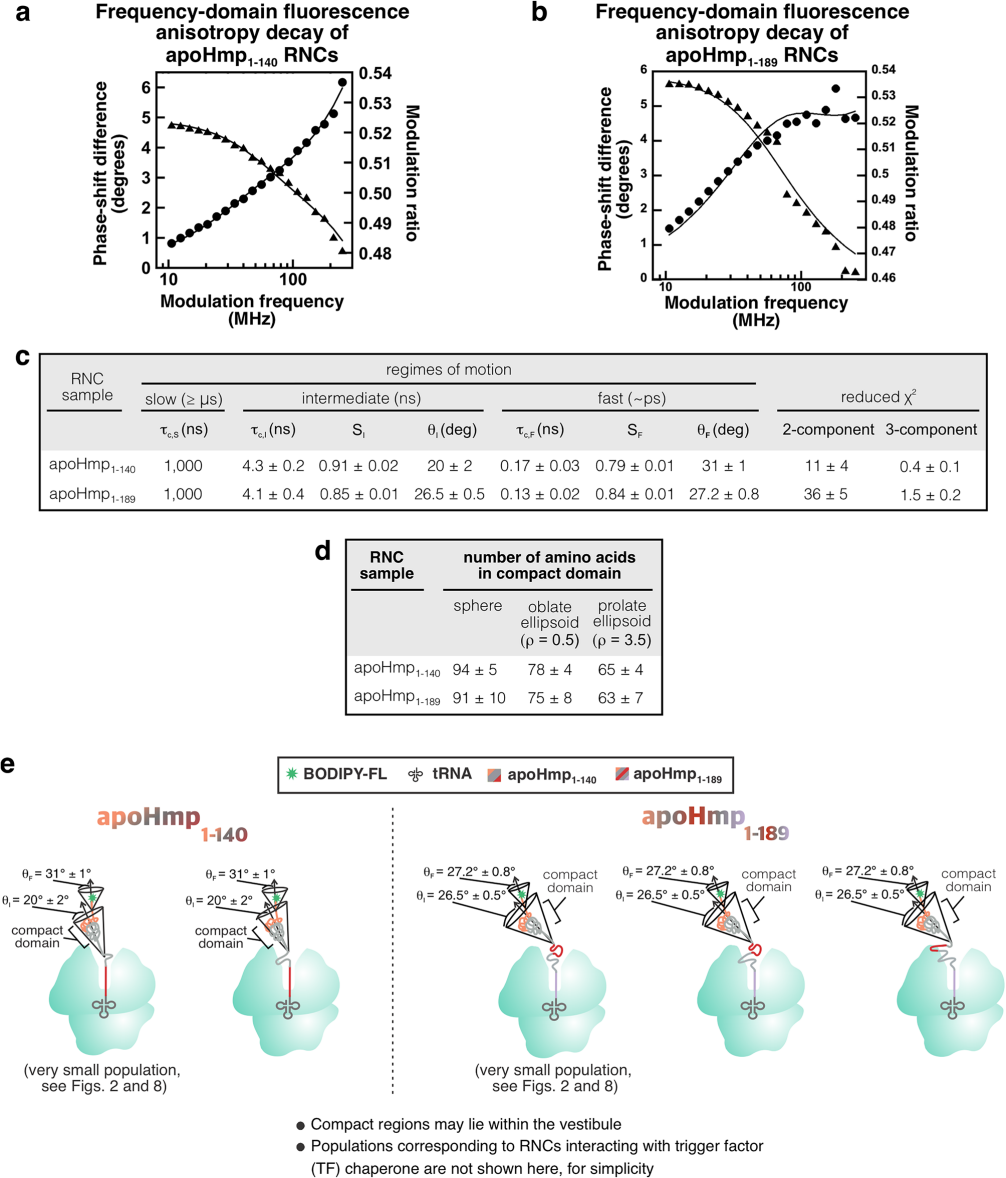
Fluorescence-anisotropy decays reveal that apoHmp nascent chains have a compact N-terminal region. Representative frequency-domain fluorescence anisotropy decay data of (**a**) apoHmp_1–140_ and (**b**) apoHmp_1–189_ RNCs. (**c**) Table summarizing anisotropy decay parameters including rotational correlation times (τ_c_), order parameters (S) and cone semi-angles (8). The S,I, and F subscripts denote slow-, intermediate-, and fast-timescale motions, respectively. Uncertainties are reported as ± SE for n = 3–5. Three-component anisotropy fits were selected as best fits if they led to a 2.5-fold (or larger) decrease in reduced ${x}^{2}$, relative to two-component fits. The ${x}^{2}$ of the chosen model is shown in bold. (**d**) Table summarizing the number of amino acids comprising the RNC compact region, deduced from the τ_C_ rotational correlation time and assuming spherical, oblate ellipsoid, or prolate ellipsoid nascent-chain shapes. The parameter p denotes the axial ratio. (**e**) Cartoon representation of apoHmp_1–140_ and apoHmp_1–189_ RNCs based on the fluorescence anisotropy decay data shown in this figure and the data in Fig. 2. The wild-type (WT) cell-free system, corresponding to the WT strain, was employed for the data shown in this Figure. This cell-free system contains the following chaperone concentrations: TF (2–15 nM), DnaK (0.5 µM), DnaJ (0.04 µM) and GrpE (0.05 µM).

Representative data for apoHmp_1–140_ and apoHmp_1–189_ are shown in panels a and b of Fig. 4, respectively. In addition, low-pH gels documenting the lack of heterogeneity in RNC length are shown in Supplementary Figure S13. Both RNCs display informative frequency-domain anisotropy decay profiles. As shown in Fig. 4c and consistent with the very low reduced χ^2^ values, the fits that include 3 rotational-tumbling components give the best results. Importantly, panels c and d of Fig. 4 show that both apoHmp_1–140_ and apoHmp_1–189_ RNCs are characterized by an N-terminal compact domain that tumbles independently from the ribosome. This conclusion was reached upon applying known procedures based on a combination of microscale viscosity and fluorescence depolarization in the frequency domain. In both cases, this domain spans ca. 63–94 residues, depending on the exact shape. Note that RNC shape assessment is beyond the scope of this work. Regardless of the actual overall morphology of the compact domains, the fact that a compact domain of identical size is observed for both apoHmp_1–140_ and apoHmp_1–189_ suggests that both constructs undergo a similar degree of partial folding on the ribosome. Surprisingly, the observed size of the compact domain of apoHmp_1–189_ RNCs is significantly smaller than the size of the entire apoHmp domain 1, which comprises 140 residues (Fig. 1c). Therefore, biosynthesis of the additional 49 C-terminal amino acids belonging to domain 2 is not sufficient to lead to complete folding of the N-terminal domain domain 1, for this protein.

In addition, cone semi-angle analysis of the fluorescence anisotropy decay data (Fig. 4c) shows that the compact domain of apoHmp_1–189_ RNCs spans a slightly wider cone semi-angle (26.5° ± 0.5°) than apoHmp_1–140_ RNCs (20° ± 0.2°), consistent with the fact that the latter construct likely projects slightly further out from the ribosomal surface than the shorter apoHmp_1–140_ construct. In all, our fluorescence anisotropy data show that the apoHmp_1–140_ and apoHmp_1–189_ nascent chains are both comparably compact and no more than partially folded, while on the ribosome, with apoHmp_1–189_ spanning a slightly wider cone semi-angle.

All the above information on fluorescence anisotropy decays is pictorially recapitulated by the cartoons of Fig. 4e. The images presented in this figure also show a variety of compact species that take into account the r-protein-interacting and non-interacting populations deduced from the SDS-PAGE and Western blotting data of Fig. 2. Interestingly, the emerging scenario bears a close resemblance to the structure-based “lazy lollipop” model recently developed for the partially compact RNC of a related globin^50^. This work was based on a combination of single-particle cryo-electron microscopy and fluorescence anisotropy decays^50^.

**Figure 5:**
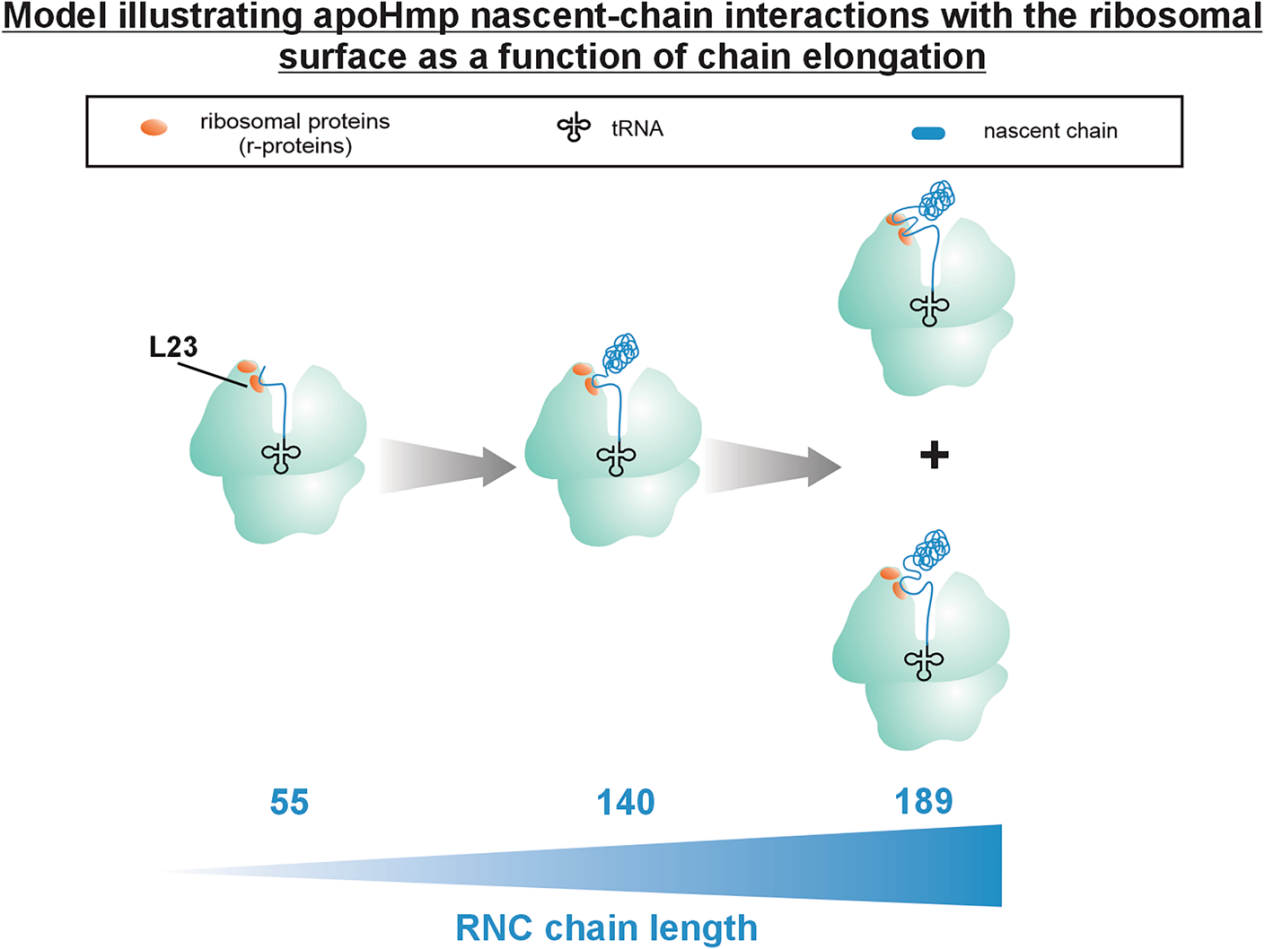
Nascent chains of the foldable protein apoHmp interact with specific ribosomal proteins in a chain-lengh-dependent fashion. Cartoon illustrating the fact that foldable nascent chains of apoHmp interact with r-protein L23 at short (55 residues) and medium-size (140 residues) chain lengths. As translation continues and nascent proteins get longer (189 residues), the interaction network extends to one additional r-protein, while preserving the approximate size of the N-terminal non-interacting compact region. This cartoon is based on data in Figs. 2 and 4.

In order to recapitulate the RNC/r-protein interaction profiles and nascent-protein conformation knowledge gained so far, a model highlighting the leading trends is shown in Fig. 5. The RNCs displayed in this figure highlight the evolution of foldable apoHmp nascent-chain interactions with r-proteins as a function of chain elongation. Briefly, when the nascent chain reaches a 55-residue length, the main detected interactions are with r-protein L23. No compact region is shown at this chain length, consistent with known fluorescence anisotropy-decay data collected on RNCs of a related globin^20^. As the nascent chain gets longer and reaches a length of 140 residues, interactions with L23 are still present, but the chain also features a non-interacting compact region that spans a cone semi-angle of ca. 20°. As the nascent chain reaches 189-residue length, two classes of RNC populations interacting with r-proteins are present. The former interacts only with L23 and the other one also interacts with an additional ribosomal protein. In both cases, an N-terminal compact region encompassing 65–94 residues is also detected. Further chain elongation and ribosome-release processes, whose investigation is beyond the scope of this work, are not illustrated in Fig. 5. These events are expected to give rise to a full-length ribosome-released folded protein, under physiologically relevant conditions.

Finally, it is useful to reflect on how the RNC compaction and amplitude of low-ns local motions, detected here, may relate to the RNC dynamics identified by other research groups via different techniques. Kaiser and coworkers detected slow (tens of seconds) conformational transitions of RNCs of a variety of single- and multi-domain proteins, by optical-tweezer technologies^90–92^. These results are consistent with the extensive interactions with the ribosomal surface detected here. However, it is hard to establish direct correlations between our data and the experiments by Kaiser and coworkers. On one hand, optical tweezers cannot directly establish the presence of RNC/r-protein interactions, and the data presented in our work only provide evidence for RNC interactions with the ribosomal surface and for N-terminal RNC dynamics. In addition, our approach is unable to detect the timescale of any intra- or inter-molecular conformational interconversions, as it is solely based on identifying local and global sub-domain rotational motions. On the other hand, our approach should be able to identify multiple slow-exchanging populations with different tumbling-motion characteristics. The fact that we do not identify multiple populations is fully consistent with the model of Fig. 4e. In this case, the slow-exchanging portion of the chain might be the one interacting with the ribosomal surface rather than the compact N terminal sub-domain, which tumbles independently on the low-ns timescale. The latter arguments also apply to a comparison between our studies and the RNC dynamics studies by the Rodnina group^31,93^. In these fluorescence-based investigations^31,93^, faster conformational interconversions (ca. 25 µs to ca. 90 ns) then in the work by Kaiser were detected, for the N-terminal domain of the HemK nascent protein. The work by the Rodnina group was carried out by fluorescence correlation spectroscopy in combination with photo-induced electron transfer (PET-FCS). The latter fluorophore/quencher-based technique relies on detecting conformational fluctuations faster than the translational dynamics of RNCs across the confocal region of the fluorescence microscope. Other studies in the Christodoulou group^6,48^, based on NMR relaxation measurements and molecular dynamics calculations, are consistent with the presence of extensive RNC/r-protein interactions detected here, as well as with intramolecular RNC conformational interconversions on the ca. millisecond timescale.

### Nascent chain-L23 complexes have the same apparent stability regardless of RNC sequence

**Figure 6:**
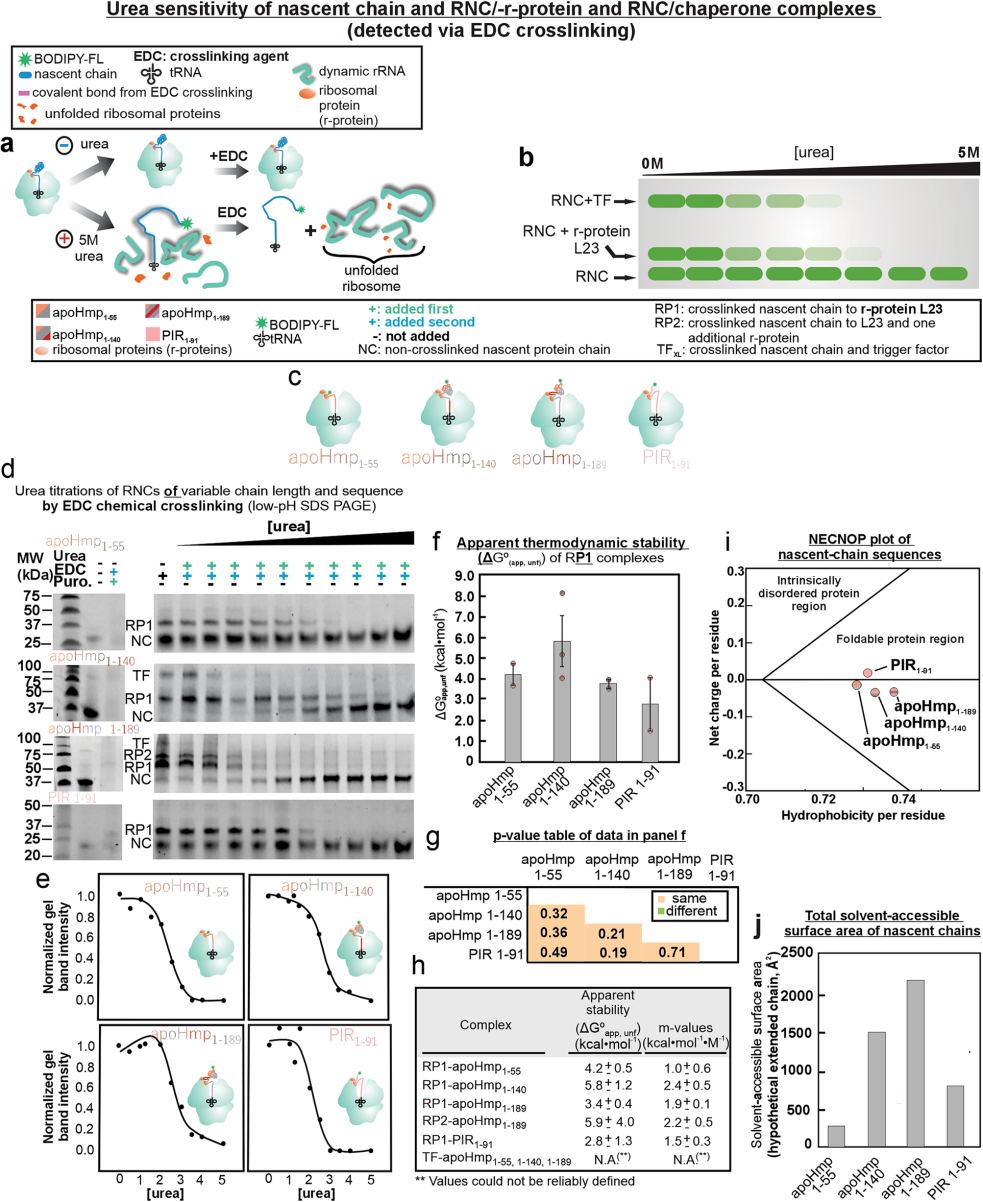
EDC-crosslinking-detected urea titrations showing the apparent stability of RNC/-r-protein complexes. (**a**) Scheme showing the expected effect of urea addition on RNCs and (**b**) corresponding low-pH SDS-PAGE gels. (**c**) Four RNCs were tested in these experiments:apoHmp_1–55_, apoHmp_1–140_, apoHmp_1–189_ and PIR_1–91_. Note that PIR_1–91_ is an intrinsically disordered protein (IDP). (**d**) Representative SDS-PAGE analysis. Gel bands are reporters of the apparent stability of complexes between RNCs and either L23 or TF. Uncropped gel images are shown in Fig. S10. (**e**) Representative urea titrations of apoHmp_1–55_, apoHmp_1–140_, apoHmp_1–189_ and PIR RNCs. (**f**) ΔG°_app,unfold_ values in the presence of low concentrations of chaperones (WT cell-free system concentrations: 2–15 nM of TF and 0.5 µM, 0.04 µM and 0.05 µM of and DnaK, DnaJ and GrpE respectively). Uncertainties are reported as ± SE for n = 2–3. (**g**) *P*-value table for a two- tailed Student’s T-test, comparing the ΔG°_app,unfold_ values of RNC/r-protein complexes. Green and orange boxes denote statistically different and statistically equivalent data, respectively, according to a 95% confidence interval. (**h**) Table displaying relevant ΔG°_app,unfold_ and m-values. (**i**) NECNOP plot^106^ displaying net charge/residue as a function of hydrophobicity/residue of PIR_1–91_, apoHmp_1–55_, apoHmp_1–140_, apoHmp_1–189_ protein chains. (**j**) Estimated total solvent-accessible surface areas of protein chains, assuming fully extended conformations. Values were computed with Surfracer^107^.

To further explore the nature of the interactions between the L23 r-protein and nascent chains of increasing length and variable sequence, we performed urea titrations with chemical crosslinking detection (Fig. 6a,b). EDC readily reacts with amines and carboxylic acid functional groups, and there is no loss of EDC reactivity even in the presence of high urea concentrations^94^. It is worth noting that the interactions identified in this work are not induced by the covalently N-terminal-linked BODIPY-502 fluorophore, as previous work has shown that this fluorophore does not interact with resuspended ribosomes under conditions like those of the present study^20^. Therefore, by unfolding the complex in the presence of urea and subsequently adding EDC, we expect to gain insights into the urea sensitivity of nascent chain-L23 complexes. While different RNC constructs are expected to bear a different number of EDC-reactive residues, denaturant titration of RNC complexes always examine the same nascent chain at variable urea concentration. Therefore, it is not necessary to normalize the data on a per-EDC-reactive-residue basis, as done in other studies^49^.

After collecting gel data on representative apoHmp and PIR nascent chains (Fig. 6c), we estimated the apparent stability (ΔG°_app,unfold_) of nascent chain-L23 complexes following a known extrapolation method which is further described in the SI methods^95^. Representative EDC-mediated urea titrations are shown in Fig. 6d. Corresponding plots and apparent-stability data are displayed in Fig. 6e,f. The matching two-tailed Student’s t-test is provided in Fig. 6g. As shown in Fig. 6h, the apparent stability values for the apoHmp and PIR nascent-chain/L23 complexes (RP1 complexes) range between ΔG^0^_app,unf_ of 2.8 ± 1.3 and 5.8 ± 1.2 kcal mol^−1^. As shown in the t-test of Fig. 6g, all complexes display the same apparent stability within error. The corresponding values for the apparent unfolding equilibrium constants K_app_ (Table S1) are within the 590 ± 340 µM to 58 ± 41 mM range. These values, if regarded as estimates of the lower limits of the expected dissociation constants of r-protein/RNC complexes, suggest that the binding affinity of the apoHmp and PIR nascent-chain/L23 complexes (RP1 complexes) is overall rather weak. This qualitative estimate is consistent with the need for the interactions to be continuously remodeled during translation elongation. Interestingly, the observed trends apply even though the nascent-chain portions emerging from the ribosomal exit-tunnel core have widely different nonpolar and net-charge-per-residue (Fig. 6i) as well as widely different total nonpolar surface accessible surface-area values (Fig. 6j).

In summary, the urea-titrations in Fig. 6 show that the urea sensitivity of r-protein-nascent-chain complexes is similar regardless of the nature and length of the nascent chain, across the short and long (55- to 189-residue) chains examined here. In other words, RNC/-r-protein complexes have the same apparent stability, even though the RNCs have widely different physical properties and compaction (as discussed above and) and in the case of PIR, lack of compaction (discussed in previous work)^49,51^. Given that the amino-acid sequences of the interacting regions of apoHmp_1–55_ and apoHmp_1–140_ must be different yet the interactions are of comparable apparent strength, the contacts are likely to be of nonspecific nature (Fig. 6 f–h). This scenario, again, is consistent with the fact RNC-r-protein interactions likely need continuous remodeling during translation elongation.

Finally, the urea titrations described in Fig. 6 are highly informative, as they also display the urea dependence of complexes between nascent chains and the trigger factor chaperone (RNC/TF complexes), e.g., see selected upper bands in Fig. 6d. The quality of the data for the RNC/TF complexes was rather poor due to unreliable pre-transition baselines, therefore we did not deduce apparent stability values. On the other hand, as shown in Fig. 6d and in the plots of Fig. S3, the complexes with the TF chaperones are consistently less stable than the corresponding complexes with the L23 protein. This result suggests that the interactions between RNCs and the TF chaperone are even weaker than the interactions between RNCs and the L23 r-protein. Hence, nascent chains interacting with TF may in general be allowed more extensive conformational sampling in their bound state than nascent chains interacting with r-proteins. Additional future work will be devoted to further explore this hypothesis.

### Nascent chain and r-protein interaction strength does not vary in the presence of one or more molecular chaperone

Next, we explored the effect of molecular chaperones TF and Hsp70 on the RNC-r-protein interactions via the same type of EDC-mediated urea titrations employed in the last section. The effect of Hsp70 was examined in the context of the K/J/E chaperone system. TF is known to associate with prokaryotic ribosomes^96^ and K/J/E works in cooperation with TF^97^ to promote nascent-protein folding and prevent nascent-protein aggregation^8,83,98,99^.

**Figure 7:**
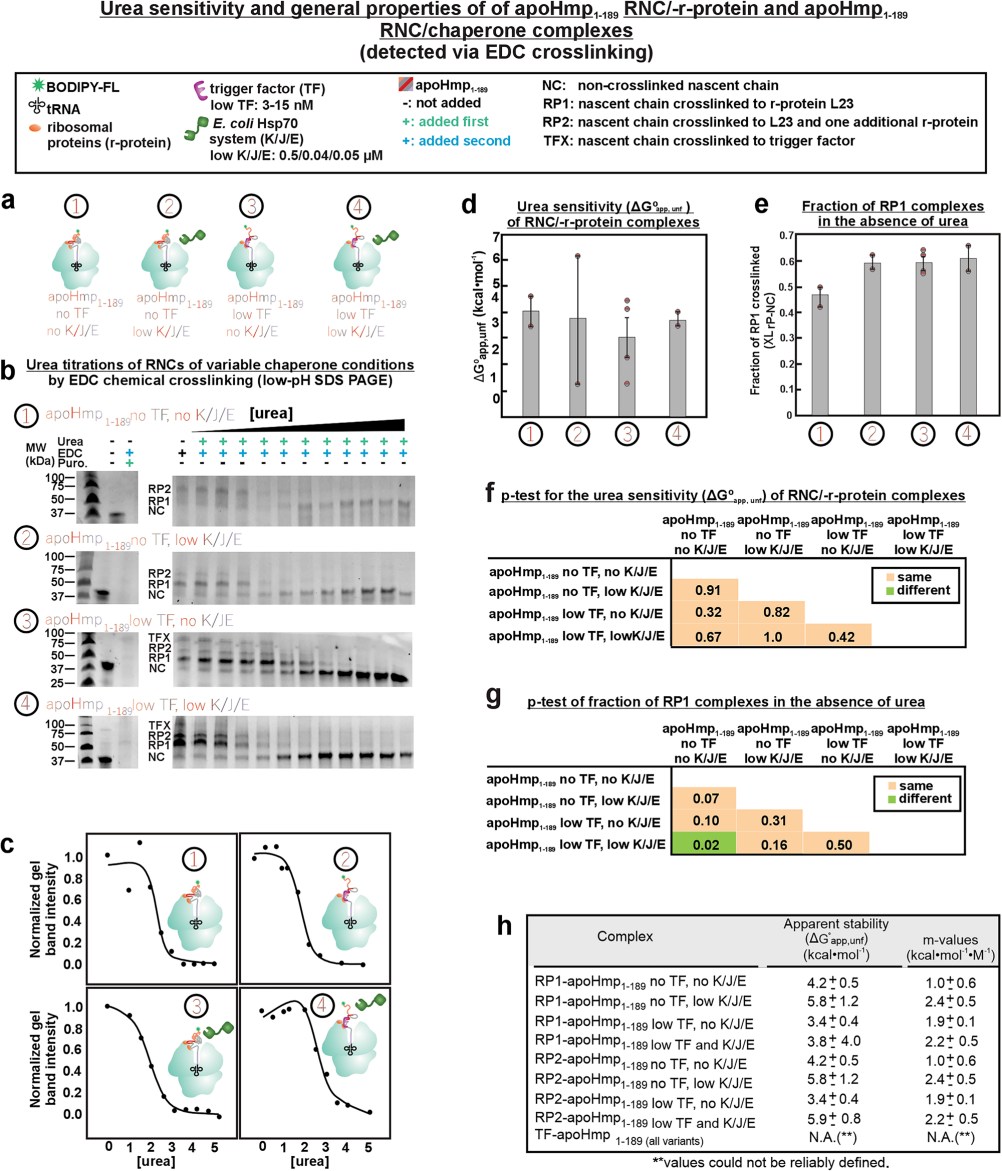
Low pH SDS-PAGE and urea-titration analysis of apoHmp1-189 in the absence and presence of TF and K/J/E chaperones. (**a**) Cartoon illustrating the tested RNCs. (**b**) Low pH SDS-PAGE analysis of complexes including apoHmp_1–189_ RNC and either r-proteins or molecular chaperones as a function of increasing urea concentration. Uncropped gel images are shown in Figure S10. (**c**) Representative urea titration curves. (**d**) ΔG°_app,unfold_ values in the absence and presence of low concentrations of molecular chaperones (WT cell-free system: 2–15 nM TF, and 0.5 µM, 0.04 µM and 0.05 µM K/J/E, respectively). Error bars denote standard error based on 2–3 experiments. (**e**) Fraction of RNC/r-protein complexes relative to total RNCs. Uncertainties are reported as ± SE for n = 2–3. (**f**) *P*-value table for two-tailed Student’s test assuming unequal variances, comparing ΔG°app,unfold values. Green and orange boxes denote statistically different and statistically equivalent data, respectively, according to a 95% confidence interval. (**g**) *P-*value table comparing fractions of RNC/r-protein complexes. Statistical assessments were similar to those listed in panel f. (**h**) Table showing ΔG°_app,unfold_ and m values of relevant complexes.

First, we evaluated apoHmp_1–189_ devoid of both TF and the Hsp70 chaperone system (K/J/E), apoHmp_1–189_ in the presence of low concentrations of TF (2–15 νM) only, apoHmp_1–189_ in the presence of low concentrations of K/J/E (0.5, 0.04 and 0.05 µM, respectively) only, and apoHmp_1–189_ in the presence of both chaperones at low concentration (Fig. 7a). Urea titrations were carried out with increasing concentrations of urea (Fig. 7b), and the intensities of the crosslinked fractions were plotted (Fig. 7c). We then obtained a ΔG°_app, unfold_ values for each of these constructs (Fig. 7d) and evaluated them with a two-tailed Student’s t-test (Fig. 7g), similarly to what done for the data in Fig. 6. Interestingly, the apparent strength of the L23-nascent chain complex was found to be statistically similar in all cases, regardless of chaperone concentration (Fig. 7f,h). Given that this effect is not due to a variation in the fraction of crosslinked nascent chains to r-proteins (Fig. 7e,f), via Western Blot analysis, we conclude that the extent of interactions between nascent chains and L23 remains similar in the absence and presence of the TF and K/J/E chaperones (Fig. 2c). This finding suggests that nascent chains interact with ribosomal L23 in a structurally similar fashion regardless of the absence or presence of chaperones.

### RNC/r-protein interactions are attenuated at high chaperone levels in a chain-length-dependent manner

**Figure 8:**
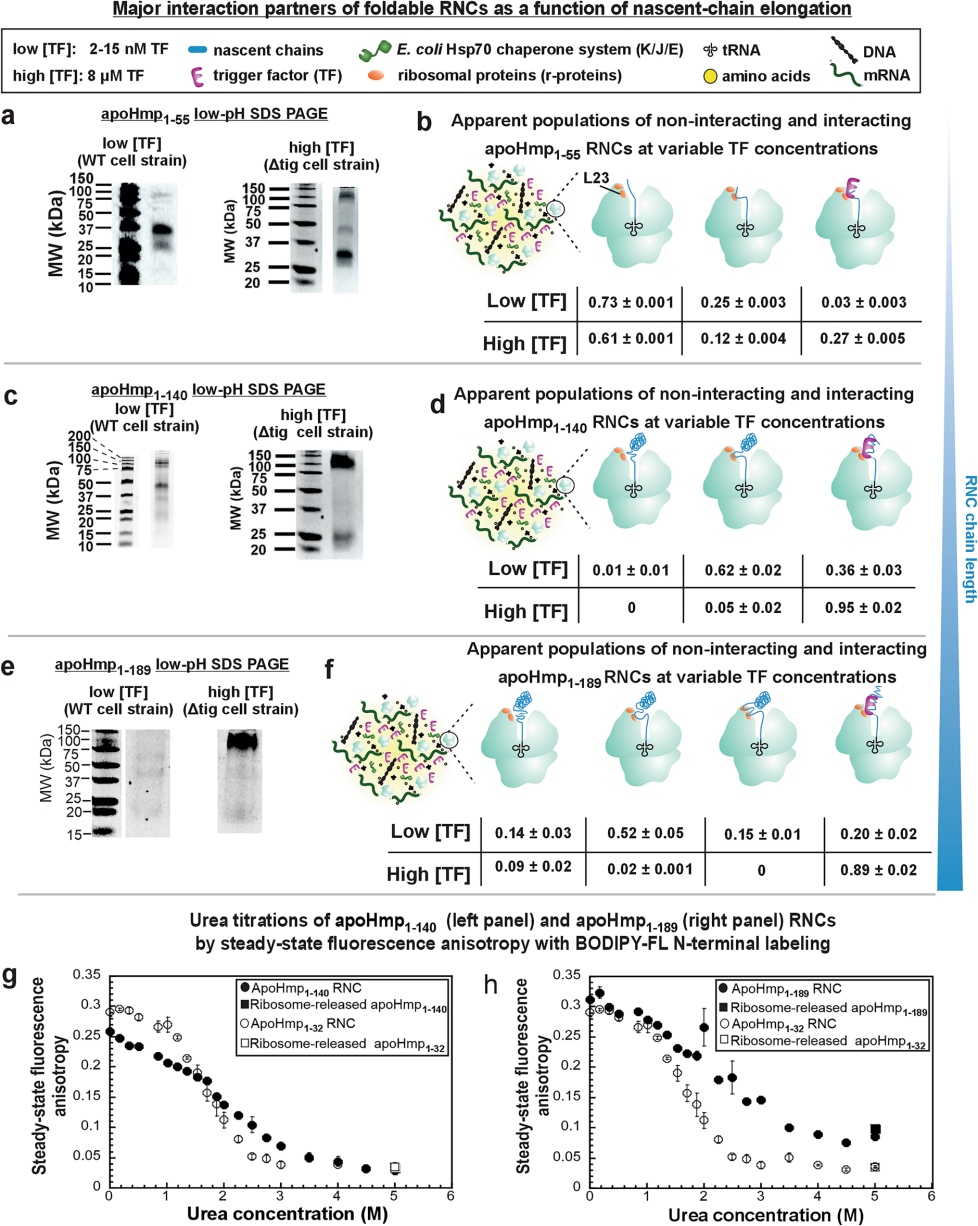
The TF molecular chaperone displaces interactions between RNCs and ribosomal proteins. (**a**) Low-pH SDS-PAGE gels showing interacting apoHmp_1–55_ RNCs after addition of EDC at low (2–15 nM) and high (8 µM) TF concentrations. (**b**) Pictorial representation of interacting apoHmp_1–55_ RNCs and their respective populations (n = 2 ± SE). (**c**) Low-pH SDS-PAGE gels showing interacting apoHmp_1–140_ RNCs after addition of EDC at low (2–15 nM) and high (8 µM) TF concentrations. (**d**) Pictorial representation of interacting apoHmp_1–140_ RNCs and their respective populations (n = 2–3 ± SE). (**e**) Low-pH SDS-PAGE gels showing interacting apoHmp_1–189_ RNCs after addition of EDC at low (2–15 nM) and high (8 µM) TF concentrations. (**f**) Pictorial representation of interacting apoHmp_1–189_ RNCs and their respective populations (n = 2–4 ± SE). The WT cell-free systems have K/J/E chaperones at 0.5, 0.04 and 0.05 µM concentrations, respectively. Uncropped gel images are shown in Figs. S10 and S11. (**g**) and (**h**) Steady-state fluorescence anisotropy (λ_exc_ = 477 nm) of apoHmp_1–140_ (**g**) and apoHmp_1–189_ (**h**) RNCs as a function of urea concentration (n = 2). The plots also include control data for the short apoHmp_1–32_ RNC, which is expected to reside entirely within the ribosomal-tunnel core (n = 2). All RNCs were selectively labeled with BODIPY-FL at the nascent-chain N terminus. RNCs were generated from a WT *E.* coli cell strain (see Methods) with no added chaperones or chaperone inhibitors. Note that the steady-state anisotropy losses of apoHmp_1–32_ RNCs at increasing urea concentration are regarded as dominated by exit-tunnel disassembly processes. The latter, in turn, lead to increased apoHmp_1–32_ RNC tumbling rates and(or) wider-amplitude local motions, resulting in decreased steady-state fluorescence anisotropies.

To further elucidate the nature of RNC/chaperone complexes, we performed experiments at low (2–15 nM) and high (8 μM) TF concentrations (Fig. 8). The high concentration values are representative of physiologically relevant TF levels upon taking into account the differences in the concentrations of actively translating ribosomes in our cell-free system and in live *E. coli* cells^49^. Interestingly, interactions with the r-protein L23 alone are mostly displaced or complemented by interactions with TF, at high TF concentration (Fig. 8). Note that, for simplicity, we show only the simplest possible representation of the interactions, in the cartoons in Fig. 8. This is because it is known from the literature that the docking site of TF on the ribosome comprises the L23 and L29 r-proteins^100^. Therefore, the L23 protein must be an interactor of TF. On the other hand, L23 may also, in addition, interact directly with the RNC. The displacement of interactions with L23 alone by interactions with TF (or by interactions with TF and L23) is more pronounced for longer RNCs, as shown by the representative gels of Fig. 8a,c,e, Fig. S2, and by the comprehensive analysis of the interacting populations shown in Fig. 8b,d,f. The shortest nascent chains of apoHmp_1–55_ only show c.a. 25% interactions with TF, even at high TF concentrations. We attribute this result to the fact that apoHmp_1–55_ is likely too short to form extensive interactions with the TF chaperone.

To summarize, in the absence of TF (Figs. 7 and S2), the nascent chain either interacts primarily with r-proteins, mainly, L23. At higher, physiologically relevant concentrations of TF (8 μM TF), RNC interactions with r-proteins are displaced (or complemented) by interactions with this molecular chaperone. It is worth noting that TF is shared with thousands of additional cellular proteins in vivo*,* unlike in the experiments shown here, which include purified resuspended RNCs. Further, our RNC concentrations are only 20–30 nM. Hence, the TF chaperone is in large excess over RNCs even at the low chaperone concentrations employed here. This scenario differs from the cellular environment where both RNCs and molecular chaperones are at comparable concentrations, within the low uM range. Therefore, we propose that the actual cellular milieu likely involves RNC populations that interact in part with TF and in part with r-proteins. In all, our findings highlight the prominent role of the ribosome as an RNC interactor.

### The presence of very short nascent chains stabilizes the 70S ribosomal complex

After exploring nascent chain and r-protein interactions, we investigated the potential effect of these contacts on the bacterial ribosome. We began by performing a series of qualitative sucrose-gradient studies on *E. coli* empty ribosomes and nascent-chain-loaded ribosomes. Our results, detailed in Figs. S4 and S5, showed that empty-70S ribosomes are more sensitive to urea denaturation than ribosomes bearing tRNAs linked to longer nascent chains. These results agree with previous sucrose gradient studies on RNCs^36^. It appears that the snc-tRNA is responsible for most of the stabilizing effect (Figs. S4 and S5). Interestingly, these data suggest that length and amino-acid sequence of the nascent protein does not influence the urea sensitivity of ribosome-RNC complexes. This study provides an interesting complement to the results by Taguchi and coworkers where RNC-dependent ribosome destabilization was observed, in the presence of translation-arrest sequences^101^.

### The peptidyl transferase center site is largely unaffected by nascent-chain sequence and length, beyond 32 residues

Next, we probed whether nascent chains of different length, amino-acid sequence and foldability affect the apparent stability of specific regions of the ribosome. We directed our focus on the peptidyl transferase center (PTC) of the *E. coli* ribosome, and we explored its urea sensitivity via a nascent-chain ribosome-release assay mediated by puromycin. These experiments employed a larger set of RNCs than in the previous sections.

The results of puromycin-release-detected urea titrations are shown in Fig. S8 and further described in the Supplementary Information. Overall, the data show that the apparent stability of the ribosomal PTC is not affected by the presence of nascent chains longer than 32 residues.

### The global urea sensitivity of ribosomal proteins is largely unaffected by nascent-chain sequence and length

Next, we explored the effect of nascent-chain properties on the overall apparent stability of r-proteins via urea titrations based on Trp fluorescence emission. Trp is a well-known fluorescent reporter, and its emission properties are highly environmentally sensitive. Urea titrations were carried out and Trp fluorescence emission was monitored (Fig. S9b,c). Spectral shifts were regarded as reporters of r-protein folding, and centers of mass of emission spectra were assessed to generate titration curves reporting on the urea sensitivity of r-proteins. Note that incubation time totaling the measurements from beginning and end of experiments did not change the spectral center of mass (Fig. S9d). Urea titration data were processed according to Santoro and Bolen^95,102^. Individual representative titration curves are shown in Fig. S9e.

The ΔG°_app, unfold_ for each construct are plotted in Fig. S9f and corresponding t-test values are tabulated in Fig. S9g. Nearly all the constructs show statistically similar results, with ΔG°_app, unfold_ values ranging from 2 to 5 kcal mol^−1^ Hence, the presence of peptidyl tRNA, regardless of nascent-chain characteristics, does not affect the urea sensitivity of r-proteins. As shown in previous sections, some nascent chains interact with the specific ribosomal protein L23. On the other hand, these interactions are not sufficiently strong to be detected via this assay, which monitors the overall sensitivity to urea of all r-proteins.

### The urea-unfolding profile of RNCs is complex and strongly chain-length-dependent

In order to more specifically monitor the RNC stability, we also explored the urea sensitivity of ribosome-bound nascent chains of apoHmp_1–140_ and apoHmp_1–189_ by steady-state fluorescence anisotropy titrations. We took advantage of the fluorescence anisotropy of the BODIPY-FL fluorophore, which is selectively linked to the RNC N terminus and senses the local and global rotational motions of the nascent chain. The results of these experiments are shown in Fig. 8 (panels g and h). The urea titration profiles are complex, and the data support a poorly cooperative (likely multi-step) unfolding mechanisms for both the apoHmp_1–140_ and apoHmp_1–189_ RNCs. Upon comparing the urea-titrations of the RNCs in Fig. 8g,h with the urea-titrations of the RNC/ribosomal-protein (r-protein) complexes of Fig. 6d–g, we attribute the second broad transition of Fig. 8g,h to the disruption of RNC/-r-protein interactions. In addition, the control titration of the very short apoHmp_1–32_ RNC, shown as a reference dataset in Fig. 8g,h (open circles), is regarded as a sensor of ribosomal-tunnel disassembly. Indeed, the short 32-residue peptide must be deeply buried within the ribosomal tunnel, and the disruption of its rotational tumbling is likely to coincide with the disruption of the RNC-loaded ribosomal tunnel. This scenario is also qualitatively consistent with the sucrose-gradient data of Supplementary Fig. S4 and S5. In addition, the fluorescence anisotropy-decay data of Fig. 4 remind us that the nascent-chain N-terminal domain comprises 63–94 residues. Taking all the above information into account, the RNC urea titrations of Fig. 8g suggest that the apoHmp_1–140_ has a very thermodynamically unstable N-terminus compact domain that unfolds before the ribosomal tunnel does. In addition, this RNC has a second more thermodynamically stable domain (likely including the interactions with the L23 r-protein shown in Fig. 2), that unfolds in concert with the disassembly of the ribosomal tunnel.

A different scenario applies to the longer apoHmp_1–189_ RNC, which has a more thermodynamically stable N-terminal domain, likely due to the larger number of residues emerging from the ribosomal tunnel, relative to apoHmp_1–140_, as shown in Fig. 8h. In the case of apoHmp_1–189_, the entire RNC undergoes a broad unfolding transition with a denaturant concentration half-point (C_1/2,urea_) of ca. 2.5 M urea. Therefore, the apoHmp_1–189_ RNC is overall more stable than the apoHmp_1–140_ nascent chain, and it unfolds as the ribosomal tunnel disassembles. In all, our RNC titration data show that the thermodynamic stability of the apoHmp RNC strongly depends on chain length and is different for different regions of the chain.

While a comparison between the stability of RNCs and corresponding released proteins is beyond the scope of this work, it is worth noting that the apoHmp_1–140_ RNC has an overall thermodynamic stability (C_1/2,urea_ = 2 M) identical to that of the native protein (C_1/2,urea_ = 2 M), under comparable solution conditions^69^. This observation is consistent with the urea titration data of Fig. 8g, indicating that the N-terminal domain is less stable than the overall ribosome-released protein (see 1st unfolding transition) while the RNC/r-protein interactions have an overall thermodynamically stabilizing effect (see 2nd unfolding transition). Interestingly, these opposite (stabilizing and destabilizing) effects balance each other nicely, so that overall protein stability is not compromised at the end of ribosome-assisted biosynthesis, even before release of the ribosome. In all, the above results are consistent with prior literature on RNC stability^5,31,35–37^, and provide additional insights.

## Conclusions

Here, we identify the ribosomal protein L23 as a specific nascent-chain-interacting partner. L23 establishes noncovalent contacts with nascent chains of the multi-domain foldable model protein apoHmp, which lacks signal/arrest sequences. As nascent chains elongate, the RNC interaction network expands to another ribosomal protein. A non-interacting N-terminal compact RNC region comprising 63–94 residues has also been identified for nascent chains bearing both 140 and 189 residues. A model recapitulating the presence of both RNC/r-protein interactions and non-interacting N-terminal regions is shown in Fig. 5. Interactions with the TF take over, at high TF chaperone concentrations. Interestingly, ribosomal-protein/nascent-chain complexes have a similar weak apparent stability regardless of nascent-chain sequence, length and degree of foldability. Therefore, we propose that r-proteins shield nascent foldable proteins from aggregation before intramolecular folding becomes thermodynamically favorable, during and(or) immediately after translation. These findings unveil the presence of interactions between a foldable nascent chain and the L23 ribosomal protein. In addition, the data reveal that these interactions coexist with nascent-chain compaction across the N-terminal region, suggesting ribosome-facilitated aggregation-prevention as well as conformational sampling.

## Materials and methods

### Preparation of empty ribosomes

Empty ribosomes were generated from an in-house**-**prepared A19 WT or A19 Δtig *E. coli* S30 cell extract as described^20,71^. Briefly, cells were grown in Luria–Bertani (LB) broth and harvested at mid-log phase (A_600_ ~ 0.6). The cells were lysed through a French press (thermo Electron Corporation, Waltham, MA) at ~ 12,000 psi with a single passage. The lysate was subject to centrifugation at 30,910 g and 20 °C for 20 min. After centrifugation, the supernatant was incubated in translation buffer (0.75 M Tris–HCl pH 8.2, 7.5 mM DTT, 21 mM Mg(OAc)_2_, 500 µM amino acids, 6 mM ATP, 67 mM PEP and 160 µg·mL^−1^ pyruvate kinase) for 80 min to remove any endogenous mRNA from ribosomes. The supernatant was then dialyzed (12–14 kDa MWCO) in buffer (10 mM Tris–HCl pH 8.2, 14 mM Mg(OAc)_2_, 60 mM KOAc and 1 mM DTT) for 12 h, with a buffer exchange every 4 h. The resulting A19 cell extract was used as the empty-ribosome sample.

### Preparation of RNCs

RNCs were generated using an in-house prepared A19 *E. coli* transcription-translation coupled cell-free system^20,71^ as described. Cell strains either including (WT) or lacking (*Δtig*) the trigger factor gene were employed^20,71^. Hsp70 chaperone activity was suppressed via the KLR-70 peptide^103^ to a final concentration of 0.2 mM. Transcription-translation proceeded for 30 min at 37 °C in the presence of BODIPY-FL-Met-tRNA^f−Met^ to specifically label RNCs at the N terminus. BODIPY-FL-Met-tRNA^f−Met^ was prepared as described^20^. RNCs were stalled at various lengths to generate the desired apoHmp and PIR constructs via oligodeoxynucleotide-directed mRNA cleavage^20,104,105^. An anti-ssrA oligonucleotide^20^ was added to a final concentration of 12.83 pmol µL^−1^ to prevent premature release of stalled RNCs. RNC pellets were isolated via a sucrose cushion (1.1 M sucrose, 20 mM tris base, 10 mM Mg(OAc)_2_, 500 mM NH_4_Cl, and 0.5 mM EDTA, 1 mM DTT, pH 7.0, as described)^20^ and subjected to ultracentrifugation at 160,000 g for 1 h at 4 °C. The purified pellet was dissolved in resuspension buffer (10 mM tris–HCl, 10 mM Mg(OAc)_2_, 60 mM NH_4_Cl, 0,5 mM EDTA and 1.0 mM DTT, pH 7.0) by shaking in an orbital shaker at 200 rpm on ice for 1 h.

### Statistics and reproducibility

Statistical data analysis was performed with Excel V. 16.70 software. Data are displayed as the mean with ± the standard error (SE), with the number of independent experiments listed in parenthesis, (e.g., n = 2). Statistically meaningful differences between sets of data were determined via the two-tailed Student t-test. Pairs of results were regarded as statistically different if bearing *P* values < 0.05.

### Other experimental procedures

Experimental details on steady-state and frequency-domain fluorescence-anisotropy, sucrose gradients, low-pH gels, puromycin assays, chemical crosslinking and urea titrations are available in the Supplementary Information.

### Supplementary Information

Supplementary Information.

## Data Availability

The data that support the findings of the study are available from the corresponding author, S.C., upon reasonable request.
