# Supplementary material for: Nascent chains derived from a foldable protein sequence interact with specific ribosomal surface sites near the exit tunnel
Source: Sci Rep. 2024 May 29;14:12324. doi: 10.1038/s41598-024-61274-1 (PMC11137106; doi:10.1038/s41598-024-61274-1)
Supplement: Supplementary file 1 — Supplementary Information. [file 41598_2024_61274_MOESM1_ESM.docx]

**SUPPLEMENTARY INFORMATION**

**Nascent Chains Derived from a Foldable Protein Sequence Interact with Specific Ribosomal Surface Sites**

**near the Exit Tunnel**

Meranda M. Masse^1^, Valeria Guzman-Luna^1^, Angela E. Varela^1,†^, Ummay Mahfuza Shapla^1,^ Rachel B. Hutchinson^1,‡^, , Aniruddha Srivastava^1,˩^ , Wanting Wei^1,⸹^, Andrew M. Fuchs^1^ & Silvia Cavagnero^1,^*

^1^  Department of Chemistry, University of Wisconsin-Madison, Madison, Wisconsin, 53706, USA.

**Present Addresses**

^†^ A.E.V.: School of Veterinary Medicine, University of Wisconsin-Madison, Madison, Wisconsin, 53706, USA.

**^‡^** R.B.H.: Department of Food Science, University of Wisconsin-Madison, Madison, Wisconsin, 53706, USA.

^˩^ A.S.: McGaw Medical Center, Northwestern University, Chicago, IL 60611.

^⸹^ W.W.: AIDS Vaccine Research Laboratory, University of Wisconsin-Madison, Madison, Wisconsin, 53711, USA.

Correspondence and requests for materials should be addressed to Silvia Cavagnero.

**SUPPLEMENTARY TEXT**

**Sucrose-gradient analysis of 70S empty ribosomes and ribosome-peptidyl-tRNA complexes.** In order to explore the effect of nascent-chain characteristics on the bacterial ribosome, we examined whether the incorporation of aminoacyl initiator tRNA (Met-tRNA^fMet^) or very short peptidyl tRNAs affects the ribosomal complex in the presence of urea. We collectively denote these species, which were generated via oligodeoxynucleotide-directed mRNA cleavage ^1-3^ (see Methods), as tRNAs carrying short nascent chains, or snc-tRNAs. Note that the antisense DNA construct used in the oligonucleotide-directed mRNA cleavage approach was designed to generate ribosome stalling after the first N-terminal residue (Met) of the nascent chain only. It is known that the *E. coli* RNAse H enzyme, employed here in conjunction with antisense oligodeoxynucleotides, typically establishes well-defined sharp cleavage sites, characterized by a site-specific distribution of the cleavage site of ca. 1-3 nucleotides ^3^. Hence a dominant population of very short (1-3 residues) nascent chains in the ribosomal complexes encompassing snc-tRNAs is expected, consistent with the observed sharp gel bands (Fig. S4g). It follows that the nascent chains belonging to snc-tRNAs are not sufficiently long to interact with ribosomal proteins (r-proteins) across the ribosomal exit tunnel ^4^. In addition, the specific thermodynamic stabilization imparted by RNA-oligodeoxynycleotide complexation was designed to be at least 12.4 kcal mol^-1^, with a corresponding antisense-oligodeoxynucleotide length ranging from 11 to 38 DNA bases. The above free-energy value is significantly larger than what was used in the original report of the oligodeoxynucleotide-directed RNA cleavage approach ^3^. Therefore, ribosomal complexes that include snc-tRNAs are expected to bear no contributions due to interactions of nascent chains with ribosomal proteins. It is worth noting, however, that in the case of some specific RNA sequences under non-optimal conditions, the cleavage-site distribution width was found to be larger than 1-2 residues ^5^. Therefore, in our case, we expect snc-tRNAs to bear only mostly 1-3 nascent-protein residues. Any hypothetical RNCs longer than 1-3 residues are expected to be poorly populated.

Once appropriate RNCs harboring snc-tRNAs were made, we set out to test the response of the ribosome against exposure to urea. Specifically, we compared the urea sensitivity of empty 70S ribosomes (Fig. S4a-b) to that of ribosomes harboring snc-tRNAs (Fig. S4c) by sucrose gradient-detected urea titrations. Sucrose gradients are able to resolve ribosomal subunits and entire 70S ribosomal particles ^6^. Further, these gradients have previously been employed to monitor the unfolding of whole ribosomes or ribosomal subunits in the absence and presence of targeted structure-perturbing buffers ^7-14^ (e.g., containing EDTA or other related agents) or classical denaturing agents like urea ^15-17^. Conveniently, high concentrations of urea do not perturb elution-profile integrity through line-broadening or other effects ^18^. Therefore, urea titrations of the 70S ribosome including sucrose-gradient detection are a powerful tool to explore ribosomal-subunit dissociation and unfolding.

Sucrose-gradient elution profiles were monitored at 260 (Fig. S4) and 280 nm (Fig. S5) in separate experiments, to probe for any potential differences in the response of rRNA and r-proteins. Our data show that rRNA and r-proteins belonging to 70S ribosomal particles devoid of tRNA and nascent chains, denoted here as empty ribosomes, are overall stable up to 1.0 M urea, with only a small extent of dissociation of the 70S particle into its 30S and 50S subunits (Fig. S4a). At 2 M urea, empty ribosomes undergo subunit dissociation accompanied by extensive line-broadening. Due to the dominant presence of intact 16S and 23S rRNA band in ethidium bromide-stained agarose gels (Fig. S6), line broadening is not ascribed to rRNA degradation. Therefore, we interpret the broad sucrose-gradient peaks observed at 2 M urea as diagnostic of rRNA and(or) r-protein unfolding accompanied by conformational heterogeneity. At > 2 M urea, both the empty ribosome, and ribosomes harboring snc-tRNA undergo severe line-broadening (Fig. S4c). At the highest urea concentration tested in this work (4 M), line-broadening is so extensive that it is difficult to deconvolute the contribution of individual ribosomal components. A similar scenario is supported by the empty-ribosome data at 280 nm, suggesting that rRNA and r-protein unfolding proceeds in concert (Fig. S5a).

Ribosomes carrying tRNAs linked to very short nascent chains (snc-tRNA) are also characterized by progressive disassembly, as urea concentration increases (Figs. S4 and S5). Unlike empty ribosomes, however, the ribosomes harboring snc-tRNAs are still ca. 50% intact at 2 M urea. Further, at this urea concentration the 30S and 50S subunits (dissociated from 70S intact ribosomes) have not yet undergone any unfolding, given that no line broadening is observed.

Notably, the sucrose gradient profiles of ribosomes harboring snc-tRNAs also display two additional peaks eluting after the 70S ribosome (Figs. S4c and S5c). These features are either due to polysomes (i.e., nearby ribosomes linked via the same mRNA strand) or to self-associated ribosomes brought together by through-space noncovalent surface contacts. The mRNA encoding short nascent-chains (sncs) is predominantly cleaved only 11 ribonucleotides away from the mRNA ribosome binding site, i.e. the Shine-Dalgarno sequence ^19^. Ribosome profiling ^20^ and structural considerations reveal that each translating bacterial ribosome spans a length corresponding ca. 24 ribonucleotides ^21^. Further, computer simulations suggested that polysomes have c.a. 24 residues between neighboring ribosomes ^22^. Therefore, geometrical factors render polysome formation very unlikely. To gain further insights, transmission electron microscope (TEM) negative stain images of ribosomes carrying snc-tRNAs were acquired in the presence of 2% methyl-tungstate ^23^. The representative TEM image displayed in Fig. S4f shows that, in addition to isolated ribosomes (within dashed blue squares), some closely spaced ribosomes (within dashed red squares) comprising 2 or more particles are also present. The spatial arrangement of these particles renders it impossible to establish where these species are polysomes or other forms of self-associated ribosomes. While polysomes seem unlikely due to the above-listed geometrical arguments, it is possible that some longer-than expected nascent chains may be populated in these samples, preventing the ruling out of polysomes. In all, the origin of the late-eluting peaks found in ribosome samples harboring snc-tRNAs remains unestablished and further future investigations are required to shed further light on this matter. On the other hand, the disassembly pattern of these peaks is entirely like that of 70S ribosomes harboring snc-tRNAs. Therefore, the late-eluting peaks do not add any new information nor modify the conclusions reached for the 70S particles.

The urea sensitivity of a representative ribosome harboring a longer nascent chain (32-residue long) derived from *E. coli* Hmp_108-140_ has also been analyzed. The results are shown in Figs. S4d,e and S5d,e. These ribosomes behave in the same way as ribosomes harboring snc-tRNAs, suggesting that nascent-chain length does not affect the apparent stability of the 70S ribosome, and that the enhanced apparent stability may be dominated by the role of the tRNA. Additional experiments probing this topic in further detail are described in some sections of the main manuscript.

In summary, the data in Figs. S4-6 show that empty-intact 70S ribosomes are more sensitive to urea-induced denaturation than ribosomes carrying tRNAs linked to nascent chains of variable lengths.

**Nascent-chain sequence and length (beyond 32 residues) does not affect the PTC.** Puromycin is a small-molecule antibiotic that induces premature release of nascent polypeptides from the ribosome. It mimics the adenosine-Phe portion of the CCA 3’ end of Phe-tRNA^Phe^ (Fig. S8a). Puromycin gets incorporated into nascent polypeptides as a result of nucleophilic attack of the carboxyl C -terminus of the nascent chain ^24^. On the other hand, this antibiotic works properly only if the A and P sites, which are entirely located within the 50S subunit, are intact ^25^. At high urea concentrations, the PTC is denatured and puromycin is no longer able to promote the release of nascent protein chains from the ribosome.

The main features of the puromycin ribosome-release assay and its urea-concentration dependence are illustrated in Fig. 8a,b. In short, RNC reactivity to puromycin is monitored via low-pH gels ^26^ as a function of increasing urea concentration.

We performed low-pH SDS-PAGE analysis on a variety of apoHmp RNCs comprising variable chain-length values (Fig. S8c,e), net charge and hydrophobicity, and degree of folding. The target nascent protein chains included snc (i.e., a 1-3 amino acid chain derived from apoHmp), apoHmp_1-32_, apoHmp_108-140_, apoHmp_1-140_ and apoHmp_1-189_. These constructs specifically enabled us to probe differences between nascent-chain sequences located within the ribosomal exit tunnel core, as well as partially and fully folded nascent-chain domains. Low-pH SDS-PAGE was used to generate peptidyl tRNA (PT) bands whose intensities were quantified as a function of increasing urea concentration. The peptidyl-puromycin band (PP) was not used to track ribosome release because of its highly environmentally sensitive fluorescence intensity. Titration curves were generated based on the relative band intensities of the PT bands of each construct, reporting on the extent of puromycin reactivity (Fig. S8c). The data were then fit to an equation relating the experimental observable at variable urea concentrations to the apparent stability (ΔG°_app, unfold_) of each construct, following the general procedures by Santoro and Bolen ^27,28^ (see Methods).

As shown in Fig. S8d-f, the results indicate that the apparent stability of the PTC center, monitored via puromycin activity assays, is very similar for all nascent-chain construct. Moreover, the two-tailed Student’s test assuming unequal variances (Welch’s test) shows that most constructs display equivalent behavior ( Fig. S8f). In other words, most constructs contribute to a similar extent to the apparent stability of the PTC, amounting to ca. 2.5 – 4.5 kcal •mol^-1^. Therefore, the urea sensitivity of the PTC does not depend on nascent-chain properties. and may be dominated by the stabilizing effect of the tRNA. The results also suggest that the specific tRNA sequence does not have an effect either. An exception is provided by snc and apoHmp_1-140_ nascent proteins, given that the apoHmp_1-140_ construct displays a statistically significant PTC-stabilizing role. This feature is likely not due to the compact partially folded state of apoHmp_1-140_, given that fluorescence anisotropy shows that nascent apoHmp_1-189_ has a comparable degree of compaction.

Note that ribosome disassembly and unfolding due to denaturing agents is an irreversible process ^29^. Therefore, true thermodynamic ΔG°_unfold_ values cannot be obtained, upon treating the ribosome with denaturing agents. Thus, we refer to the ΔG°_unfold_ values derived in this and the following sections of this work as apparent stability values. The mere function of these quantities, which were derived from data fitting of urea-titrations, is to describe the urea sensitivity – and not the thermodynamic stability – of specific components of *E. coli* ribosomes.

**Sequence of events leading to ribosome disassembly: role of tRNA and nascent proteins.** Finally, a proposed equilibrium ribosome disassembly mechanism based on the data of Figs. S4 through S6 and Figs. 9 and 10 is shown in Fig. S7. The simple steps displayed in panel a of Fig. S7 pertain to empty 70S ribosomes and are consistent with the sucrose gradient data. The ribosome starts disassembling into its component 50S and 30S subunits at 1 M urea. More extensive subunit disassembly together with subunit unraveling follows at higher urea concentrations. This process is accompanied by pervasive r-protein and rRNA conformational heterogeneity, likely due to disruption of secondary and tertiary structure leading to r-RNA and r-protein unfolding. Panel b of the same figure shows how the process gets modified if the ribosome carries aminoacyl or peptidyl tRNA. Briefly, in this case the ribosomal-subunit disassembly occurs at higher urea concentrations (1 -2 M). Given the weak dependence of the sucrose-gradient and puromycin assays on the nature of the nascent chain, we deduce that the tRNA likely dominates the effect and that the length, hydrophobicity, net charge and foldability of apoHmp nascent chains do not play a stabilizing role in ribosome stability.

**SUPPORTING MATERIALS AND METHODS**

**Generation of RNC Complexes for Anisotropy Data Collection:** Cell-free transcription-translation was used to generate 100 uL of apoHmp_1-140_ or apoHmp_1-189_ RNC samples. These complexes were purified upon centrifugation (160,000 rcf for 60 min at 4 °C) over 150 μL of sucrose cushion (1.1 M sucrose, 20 mM tris-HCl, 10 mM magnesium acetate, 500 mM ammonium chloride, 0.5 mM EDTA, and 1 mM dithiothreitol, pH adjusted to 7.0). Pellets containing RNC complexes were resuspended in 20 μL of resuspension buffer (10 mM Tris HCl, 10 mM magnesium acetate, 60 mM ammonium chloride, 0.5 mM EDTA, and 1 mM dithiothreitol, pH 7.0). These procedures are similar to previously published methodologies^1^.

**Fluorescence Anisotropy Decay: Data Collection and Analysis.** Fluorescence anisotropy-decay data were collected with a Chronos frequency-domain fluorometer (ISS Inc.) as previously described ^30,31^. Samples were excited with a 477 nm laser diode. A 480 ± 5 nm band-pass filter and a 495 nm long-pass filter (Chroma Technology) were used for the excitation and emission channels, respectively. The emission polarizer was set to a 54.7° angle for lifetime measurements. The excitation polarizer was set to vertical for lifetime and anisotropy measurements. Samples were incubated at 25 °C for at least 30 min before fluorescence measurements. A circulating water bath maintained the sample temperature at 25 ± 0.1 °C during measurements.

Lifetime and anisotropy decay data were analyzed with the Globals software package (Laboratory for Fluorescence Dynamics, LFD).^32^ Reduced χ^2^ values were calculated using a standard deviation of 0.2° for the phase and 0.004 for the modulation.^33^ Lifetime data were fit with a three-component exponential decay including one lifetime component fixed to 1 ps to account for light scattering, and two lifetimes for the fluorophore, which were allowed to float to determine the best fit. The frequency-dependent G-factor was determined experimentally on the same day as the anisotropy decay measurements, and this parameter was used to correct the anisotropy values. The anisotropy data were then fit to multiexponential decays, with the fundamental anisotropy r_0_ fixed to 0.37. The χ^2^ values of the two and three-component decays were compared to determine the best fit for the anisotropy data. Three-component fits were selected if their χ^2^ values were ≥ 2.5 times smaller than the χ^2^ values for the two-component fits. Order parameters and cone semi-angles were calculated as described previously^31^.

**Calculation of number of amino acids in compact region.** The size of the compact region of the RNCs was calculated as described previously^30^. Spherical, oblate ellipsoid (representative axial ratio = 0.5), and prolate ellipsoid (representative axial ratio = 3.5) models were used to calculate the number of amino acids in the compact domain from the intermediate timescale rotational correlation time using eqs. 1-2.

$\#aa_{sphere}= \frac{\tau_{c,I}RTd}{\eta MW_{aa}}$ (1)

$\#aa_{ellipsoid}= \frac{\tau_{c,I}RTd (2C_{\parallel}+{4C}_{\perp})}{6\eta MW_{aa}}$ (2)

where τ_c,I_ is the intermediate timescale rotational correlation time, R is the universal gas constant, T is temperature in Kelvin, d is the hydrated protein density, η is the solution viscosity, and MW_aa_ is the average molecular weight for an amino acid (110 g/mol). We estimated the hydrated protein density considering that a standard protein dry volume of 0.75 mL/g includes 0.2 mL of water per g of protein^34^. Therefore, the hydrated protein density was estimated to be 1.05 g/mL. We used the viscosity of a solution of resuspended sperm whale apomyoglobin RNCs (1.08 ± 0.02 mPa$\cdot$s) for these calculations^30^. $C_{\parallel}$and $C_{\perp}$ are related to parallel and perpendicular components of the rotational diffusion coefficient for an ellipsoid, as defined in eqs. 3-4.

$D_{\parallel}=\left( \frac{3\rho(\rho-b)}{2(\rho^{2}-1)} \right)D_{sphere}=C_{\parallel}D_{sphere}$ (3)

$D_{\perp}=\left( \frac{3\rho\left[ \left( 2\rho^{2}-1 \right)b-\rho) \right]}{2(\rho^{4}-1)} \right)D_{sphere}=C_{\perp}D_{sphere}$ (4)

where $\rho$ is the axial ratio and b is defined as

$b=\left( \rho^{2}-1 \right)^{-1/2}ln[{\rho+\left( \rho^{2}+1 \right)}^{1/2}]$ for prolate ellipsoids (5)

$b=\left( {1-\rho}^{2} \right)^{-1/2}arctan\left[ \frac{\left( 1-{\rho^{2})}^{1/2} \right)}{\rho} \right]$ for oblate ellipsoids (6)

The derivation of equations 1-4 is reported by Hutchinson *et al*^30^*.*

**Urea-dependence RNC titrations followed by steady-state fluorescence anisotropy: data collection and analysis.**  Steady-state fluorescence anisotropy data were acquired at 25 °C on a PC1 fluorimeter (ISS, Urbana Champaign, IL). The excitation wavelength was 477 nm, and a 495 nm long-pass filter (HHQ495lp, Chroma Technology Corporation, Bellows Falls, VT) was used in the emission channel. The slit widths were set to 0.5 mm (corresponding to a 4 nm bandwidth) on both the excitation and emission channels. To deduce anisotropies, polarized intensity data were collected and averaged over a 238 s time window. Samples were pre-equilibrated for at least 20 min at 25 °C prior to data collection. The G factor was measured once for each experimental set, and G factor corrections were applied after raw-data collection and background subtraction^34^.

**Denaturation of ribosome-nascent-chain complexes and empty ribosomes.** A 10 M stock solution of 0.22 µm-filtered urea was prepared in resuspension buffer and the refractive index was measured with an Abbe Refractometer (Thermo Spectronic, Fisher Scientific) to derive actual urea concentrations as described ^35^. RNCs subject to sucrose cushion ultracentrifugation (see section on RNC preparation) or empty ribosomes obtained from crude S30 were incubated in the presence of variable concentrations of urea for 1 hr at ambient temperature in the dark.

**Assessment of urea sensitivity of 70S ribosome and RNCs via sucrose-gradient analysis**. An in-house prepared A19 *E. coli* mixture (with 70S ribosomes) was incubated for 1 hr at variable urea concentrations at ambient temperature. Samples were loaded onto a 5-45% sucrose gradient and centrifuged using a Beckman L-70 Ultracentrifuge with a SW41 rotor at 288, 000 x g for 1.5 hrs at 4 °C. Gradient profiles were obtained on a Biocomp Fractionator at 0.2 mm •sec^-1^. The absorbance was measured at 254 nm and 280 nm using a Triax flow cell from BioComp to check for intactness of the rRNA and r-proteins, respectively. RNCs were treated in a similar manner after denaturation in urea for 1 hr (see RNC denaturation in Methods). The 30S, 50S and 70S subunits were collected in both instances and loaded on a 2% agarose gel (0.02 M Tris Base, 0.01 M acetic acid and 0.0005 M EDTA, or 0.5x TAE). Ethidium bromide was added to a final concentration of 0.5 µg •mL^-1^. Samples were run for 100 min at 3.92 V •cm^-1^. The gel was imaged on a GE FLA 9500 Laser Imager at PMT values between 500-700.

**Imaging of RNCs via negative staining.** RNC samples prepared in the presence of Met-tRNA^fMet^  (2 mL) were placed onto a glow-discharged copper 300-mesh formvar-carbon grid (made in house by Medical Sciences Electron Microscopy staff at UW-Madison), blotted with filter paper and allowed to dry. A Nano-W staining solution (Nanoprobes) was placed on the grid in equal volume, blotted with filter paper and allowed to dry. Images were collected on a CM120 transmission electron microscope (Philips) at 140000x magnification and 80 keV using a BIOSPR12 camera.

**Assessment of apparent stability of RNC/-r-protein and RNC/chaperone complexes via a crosslinking assay.** The EDC crosslinker is capable of capturing interactions involving RNCs and r-proteins or chaperones within the ribosomal exit-tunnel vestibule and outside the ribosomal exit-tunnel core ^36^. Either ribosome-bound or ribosome-released control samples (with EDC added after RNC ribosome release) were used to probe RNC production and to test the lack of ribosome-released nascent-chain crosslinking to other species. After incubation in urea for 1 hr, a 10x concentration of EDC solution (800 mM EDC, pH 6.8-7.0) was added to RNCs to a final concentration of 1x. Samples were incubated for 30 min at 30 °C and then quenched with a 10x concentration of Quenching Buffer (1.0 M Tris-HCl pH 7.0, 1.0 M Glycine, 1.0 M KOAc) to a final concentration of 1x (0.1 M Tris-HCl pH 7.0, 0.1 M Glycine, 0.1M KOAc). Samples were loaded onto a low-pH SDS-PAGE gels using either a 10% acrylamide gel (apoHmp_1-55_, apoHmp_1-140_ and apoHmp_1-189_) or a 9% acrylamide gel (PIR) in a 1:1 ratio with loading buffer. Gels ran at 3.92 V •cm^-1^ for either 4 hours (apoHmp_1-55_, apoHmp_1-140_ and apoHmp_1-189_) or 2.5 hrs (PIR) and were imaged on a GE FLA 9500 Laser Imager. Fluorophores were excited at 473 nm, with a PMT value within the 700-950 range.

Crosslinked band intensities were evaluated via the ImageJ software ^37^. The normalized intensity of each band was assessed via relation $I_{XL}= \frac{I_{1, XL}}{I_{2,XL}} ,$ where I_XL_ is the normalized intensity of an individual crosslinked species, I_1, XL_ is the intensity of the individual crosslinked species, I_2, XL_ is the intensity of the species at 0 M urea. The fractional band intensities were then plotted as a function of urea concentration and then plotted to fit pre- and post- transition region baselines. A 2-state unfolding expression taking pre- and post-transition baseline slopes into account ^27^ was used to fit the raw urea-titration data and deduce m-value and ΔG°_H2O_ values via the Kaleidagraph software ^28^.

**Western blot analysis of r-protein interactions with L23, L24 and L29.** Western blots were performed as described ^36^. RNCs crosslinked and separated via low pH SDS-PAGE gels were transferred to a PVDF membrane. Membranes were then probed with the corresponding rabbit anti- *E. coli* monoclonal antibody at a dilution of 1:2000 for one hour at room temperature on a rocking platform, washed with TBST and further exposed to goat anti-rabbit secondary antibodies. Aliquots of the rabbit anti-uL23 antibody were kindly donated by Shu-ou Shan (California Institute of Technology). The anti-uL23 antibody was generated by GenScript, using the CGKVKRHGQRIGRRS peptide as epitope and has been validated in previous work ^38^. Rabbit anti -uL24, -uL29, and -uL23 antibodies were kindly facilitated by Bryan W. Davies (University of Texas-Austin) and Melanie Oakes (University of California, Irvine) who obtained them from Masayasu Nomura (University of Wisconsin-Madison). The antibodies were generated using the purified *E. coli* ribosomal proteins L23, L24, and L29 ^39^. Note that throughout this work nascent chains bound to tRNA in Western blots have been referred to as RNCs. While this is technically not accurate since there is no ribosome present in the gel band, RNCs are directly added to the gel where heat and SDS separate the ribosome and tRNA. This could also account for some of the smaller molecular weight bands seen throughout Western blotting that do not correspond with RNCs, either as r-protein monomers, or as apparent multimers of r-proteins have been observed previously^40^.

**Assessment of apparent stability of PTC via a puromycin-assisted nascent-chain release assay.** To confirm that the polypeptide was attached to the ribosome, a low-pH SDS-PAGE^26^ using a 9% acrylamide gel was conducted before (positive control) and after treatment of 1 mM puromycin, which reacted with the samples for 30 minutes at 37 °C. Samples were loaded on gels in a 1:1 ratio with loading buffer. Identical samples of the positive control and puromycin-released samples at 2.23 M urea were loaded on each gel to control for intrinsic gel differences (i.e., gel crosslinking, which may affect the fluorophore quantum yield). Prior to gel loading, samples were heated at 37 °C for five min and allowed to sit at room temperature for 5 min. Gels ran at 3.92 V •cm^-1^ for 3.5 hr and imaged on a GE FLA 9500 Laser Imager. Fluorophores were excited at 473 nm and a PMT value between 315-700.

Due to the number of samples, 2 gels were needed for the positive control samples and 2 gels were needed for the puromycin-released samples per experiment. Fluorescence intensity adjustments were made between gels by normalizing the 2.23 M urea samples from each gel via Equation 1

$I_{\mathrm{intergel}}=\frac{I_{2.23 M urea, gel 1}}{I_{2.23 M urea, gel 2}}*I_{x, gel 2}$ (S1)

where I_2.23 M urea, gel1_ and I_2.23 M urea, gel2_ refer to the band intensities of the 2.23 M urea sample in each gel. I_gel2_ is the band intensity of the sample loaded onto the second gel and I_x,intergel_ is any given band intensity on the second gel in comparison to the band intensity of the first gel.

To control for differences in RNC concentrations and compare band intensities between the bound and released sample gels, Equation 2 was used

$I_{\mathrm{relative}}=\frac{I_{+puro}}{I_{-\mathrm{puro}}} ,$ (S2)

where I _+puro_ is the band intensity of the puromycin-released sample and I _-puro_ is the band intensity of the positive control. Band intensities were then divided by the band intensity of the sample containing the highest urea concentration to normalize intensities between 0 and 1. The resulting intensities were plotted to fit pre- and post- transition region baselines. Using an extrapolation method ^27^, the transition region slope and y-intercept were deduced to obtain the m-value and ΔG°_H2O_ with the Kaleidagraph software ^28^.

**Ribosomal-protein (r-protein) stability assessment via a Trp fluorescence-emission assay.** The *E. coli* r-proteins collectively contain 32 Trp residues. Trp is sensitive to changes in its environment and exposure to polar solvents causes a red-shift in its excitation spectra, which was monitored as a function of increasing urea concentrations ^41-43^. RNC samples were excited at 285 nm (bandpass of 4 nm) and the fluorescence was monitored from 295-500 nm (bandpass of 4 nm) on a Photon Counting Spectrofluorimeter (ISS) since the indole group of tryptophan is the major component of UV absorbance in that region ^44-54^.

To generate a titration curve for the RNC complex, the buffer spectra was first subtracted from the produced emission spectra. Then a baseline correction was done on the resulting spectra.

The spectral center of mass of the resulting spectra was obtained using the emission spectra between 300-385 nm (to omit the scatter peak and Raman peak) and Equation 3

$Spectral center of mass \left( \mathrm{nm} \right)=\frac{\Sigma\left( \lambda\right) \times I_{\lambda}}{\Sigma\left( I \right)} ,$ (S3)

where λ is the wavelength and I_λ_ is the intensity at a specific wavelength.

The fraction of unfolded protein at each concentration of urea was determined according to

$\Delta Fraction of unfolded ribosome=\left( 1+ Q\left( \frac{\lambda_{x}-\lambda_{\mathrm{unfold}}}{\lambda_{\mathrm{fold}}-\lambda_{x}} \right) \right)^{-1} ,$ (S4)

where λ_fold_ is the shortest wavelength calculated from Equation 3 for the folded species and λ_unfold_ is the longest wavelength calculated from Equation 3 for the unfolded species. Q denotes the ratio between the quantum yields of folded and unfolded states. Quantum-yield changes were calculated by taking highest intensity values from each folded sample (0.0 M, 0.13 M and 0.45 M urea) as well as each unfolded sample (5.5 M, 6.0 M and 6.5 M urea). The three folded and unfolded values were averaged amongst their respective groups to determine Q. ΔCoM was plotted as a function of urea concentration and the pre- and post- transition baselines were determined in Microsoft Excel. Free energy of unfolding curves were generated with the software Kaleidagraph (Synergy Software) using a known extrapolation method.

**Supplementary References**

1 Ellis, J. P., Bakke, C. K., Kirchdoerfer, R. N., Jungbauer, L. M. & Cavagnero, S. Chain dynamics of nascent polypeptides emerging from the ribosome. *ACS Chem. Biol.* **3**, 555-566, (2008).

2 Behrmann, M. *et al.* Requirements for the translocation of elongation-arrested, ribosome-associated OmpA across the plasma membrane of Escherichia coli. *J. Biol. Chem.* **273**, 13898-13904, (1998).

3 Donis-Keller, H. Site specific enzymatic cleavage of RNA. *Nucleaic Acid Research* **7**, 179-192, (1979).

4 Wilson, D. N. & Beckmann, R. The ribosomal tunnel as a functional environment for nascent polypeptide folding and translational stalling. *Curr. Opin. Struct. Biol.* **21**, 274-282, (2011).

5 Günzl, A., Palfi, Z. & Bindereif, A. Analysis of RNA–protein complexes by oligonucleotide-targeted RNase H digestion. *Methods* **26**, 162-169, (2002).

6 Dos Santos, R. F. A., Cecília M & Andrade, J. M. in *RNA Chaperones* 299-310 (Springer, 2020).

7 Robinson, A. & Sykes, J. A comparison of the unfolding and dissociation of the large ribosome subunits from Rhodopseudomonas· spheroides NCIB 8253 and Escherichia coli MRE 600. *Biochem. J.* **133**, 739-747, (1973).

8 Hosokawa, K. Binding of 5 S ribosomal ribonucleic acid to the unfolded 50 S ribosomes of Escherichia coli II. *J. Biol. Chem.* **245**, 5880-5887, (1970).

9 Tal, M. Metal ions and ribosomal conformation. *Biochimica et Biophysica Acta (BBA)-Nucleic Acids and Protein Synthesis* **195**, 76-86, (1969).

10 Natori, S., Maruta, H. & Mizuno, D. i. Unfolding of Escherichia coli ribosomes by phosphate ion in the presence of oligonucleotides. *J. Mol. Biol.* **38**, 109-119, (1968).

11 Weller, D. L., Shechter, Y., Musgrave, D., Rougvie, M. & Horowitz, J. Conformational changes in Escherichia coli ribosomes at low magnesium ion concentrations. *Biochemistry* **7**, 3668-3675, (1968).

12 Beller, R. J. & Lubsen, N. H. Effect of polypeptide chain length on dissociation of ribosomal complexes. *Biochemistry* **11**, 3271-3276, (1972).

13 Belitsina, N. & Spirin, A. Studies on the structure of ribosomes: IV. Participation of aminoacyl-transfer RNA and peptidyl-transfer RNA in the association of ribosomal subparticles. *J. Mol. Biol.* **52**, 45-55, (1970).

14 Ron, E. Z., Kohler, R. E. & Davis, B. D. Magnesium ion dependence of free and polysomal ribosomes from Escherichia coli. *J. Mol. Biol.* **36**, 83-89, (1968).

15 Roberts, M. E. & Walker, I. Structural studies on Escherichia coli ribosomes: III. Denaturation and sedimentation of ribosomal subunits unfolded in urea. *Biochimica et Biophysica Acta (BBA) - Nucleic Acids and Protein Synthesis* **199**, 184-193, (1970).

16 Samelson, A. J., Jensen, M. K., Soto, R. A., Cate, J. H. D. & Marqusee, S. Quantitative determination of ribosome nascent chain stability. *Proc. Natl. Acad. Sci. U. S. A.* **113**, 13402-13407, (2016).

17 Spitnik-Elson, P. & Greenman, B. The detachment of ribosomal proteins by urea: evidence for non‐electrostatic RNA‐protein interaction in the ribosome. *FEBS Lett.* **17**, 187-192, (1971).

18 Hung, H.-C. & Chang, G.-G. Multiple Unfolding Intermediates of Human Placental Alkaline Phosphatase in Equilibrium Urea Denaturation. *Biophys. J.* **81**, 3456-3471, (2001).

19 Shine, J. & Dalgarno, L. The 3′-terminal sequence of Escherichia coli 16S ribosomal RNA: complementarity to nonsense triplets and ribosome binding sites. *Proc. Natl. Acad. Sci. U. S. A.* **71**, 1342-1346, (1974).

20 Mohammad, F., Green, R. & Buskirk, A. R. A systematically-revised ribosome profiling method for bacteria reveals pauses at single-codon resolution. *Elife* **8**, e42591, (2019).

21 Yusupova, G., Jenner, L., Rees, B., Moras, D. & Yusupov, M. Structural basis for messenger RNA movement on the ribosome. *Nature* **444**, 391-394, (2006).

22 Brandt, F. *et al.* The Native 3D Organization of Bacterial Polysomes. *Cell* **136**, 261-271, (2009).

23 Oliver, R. M. in *Methods Enzymol.* Vol. 27 617-672 (Elsevier, 1973).

24 Yarmolinsky, M. B. & Gabriel, L. Inhibition by puromycin of amino acid incorporation into protein. *Proceedings of the National Academy of Sciences* **45**, 1721-1729, (1959).

25 Wohlgemuth, I., Beringer, M. & Rodnina, M. V. Rapid peptide bond formation on isolated 50S ribosomal subunits. *EMBO reports* **7**, 699-703, (2006).

26 Kirchdoerfer, R. N., Huang, J. J. T., Isola, M. K. & Cavagnero, S. Fluorescence-based analysis of aminoacyl- and peptidyl-tRNA by low-pH sodium dodecyl sulfate-polyacrylamide gel electrophoresis. *Anal. Biochem.* **364**, 92-94, (2007).

27 Santoro, M. M. & Bolen, D. W. Unfolding free-energy changes determined by the linear extrapolation method.1.unfolding of phenylmethanesulfonyl alpha-chymotrypsin using different denaturants. *Biochemistry* **27**, 8063-8068, (1988).

28 Pace, C. N. Measuring and increasing protein stability. *Trends Biotechnol.* **8**, 93-98, (1990).

29 Bonincontro, A. *et al.* Differential stability of E. coli ribosomal particles and free RNA towards thermal degradation studied by microcalorimetry. *Biophys. Chem.* **75**, 97-103, (1998).

30 Hutchinson, R. B., Chen, X., Zhou, N. & Cavagnero, S. Fluorescence anisotropy decays and microscale-volume viscometry reveal the compaction of ribosome-bound nascent proteins. *J. Phys. Chem. B* **125**, 6543-6558, (2021).

31 Ellis, J. P., Culviner, P. H. & Cavagnero, S. Confined dynamics of a ribosome-bound nascent globin: Cone angle analysis of fluorescence depolarization decays in the presence of two local motions. *Protein Sci.* **18**, 2003-2015, (2009).

32 Beechem, J. M. & Gratton, E. in *Time-Resolved Laser Spectroscopy in Biochemistry* (ed J.R. Lakowicz) 70-81 (1988).

33 Ross, J. A. & Jameson, D. M. Time-resolved methods in biophysics. 8. Frequency domain fluorometry: applications to intrinsic protein fluorescence. *Photochem. Photobiol. Sci.* **7**, 1301-1312, (2008).

34 Lakowicz, J. R. *Principles of Fluorescence Spectroscopy*. 3rd edn, (New York : Plenum Press, 2006).

35 Warren, J. R. & Gordon, J. A. On the refractive indices of aqueous solutions of urea. *The Journal of Physical Chemistry* **70**, 297-300, (1966).

36 Guzman-Luna, V., Fuchs, A. M., Allen, A. J., Staikos, A. & Cavagnero, S. An intrinsically disordered nascent protein interacts with specific regions of the ribosomal surface near the exit tunnel. *Commun. Biol.* **4**, 1-17, (2021).

37 Schneider, C. A., Rasband, W. S. & Eliceiri, K. W. NIH Image to ImageJ: 25 years of image analysis. *Nat. Methods* **9**, 671-675, (2012).

38 Wang, S. *et al.* The molecular mechanism of cotranslational membrane protein recognition and targeting by SecA. *Nat. Struct. Mol. Biol.* **26**, 919-929, (2019).

39 Lindahl, L. *et al.* Organization of ribosomal-protein genes in escherichia-coli. 7. mapping of ribosomal-protein genes by invitro protein-synthesis using dna fragments of lambda-fus3 transducing phage dna as templates. *J. Biol. Chem.* **252**, 7365-7383, (1977).

40 Dzionara, M., Kaltschmidt, E. & Wittmann, H. Ribosomal proteins, XIII. Molecular weights of isolated ribosomal proteins of Escherichia coli. *Proc. Natl. Acad. Sci. U. S. A.* **67**, 1909-1913, (1970).

41 Beechem, J. M. & Brand, L. Time-resolved fluorescence of proteins. *Annual review of biochemistry* **54**, 43-71, (1985).

42 Demchenko, A. P. *Ultraviolet spectroscopy of proteins*. (Springer Science & Business Media, 2013).

43 Weber, G. Fluorescence-polarization spectrum and electronic-energy transfer in tyrosine, tryptophan and related compounds. *Biochemical Journal* **75**, 335, (1960).

44 Teale, F. & Weber, G. Ultraviolet fluorescence of the aromatic amino acids. *Biochemical Journal* **65**, 476, (1957).

45 Woolhead, C. A., McCormick, P. J. & Johnson, A. E. Nascent membrane and secretory proteins differ in FRET-detected folding far inside the dribosome and in their exposure to ribosomal proteins. *Cell* **116**, 725-736, (2004).

46 Ullers, R. S. *et al.* Interplay of signal recognition particle and trigger factor at L23 near the nascent chain exit site on the Escherichia coli ribosome. *The Journal of cell biology* **161**, 679-684, (2003).

47 Eisner, G., Moser, M., Schäfer, U., Beck, K. & Müller, M. Alternate Recruitment of Signal Recognition Particle and Trigger Factor to the Signal Sequence of a Growing Nascent Polypeptide. *J. Biol. Chem.* **281**, 7172-7179, (2006).

48 Peterson, J. H., Woolhead, C. A. & Bernstein, H. D. The Conformation of a Nascent Polypeptide Inside the Ribosome Tunnel Affects Protein Targeting and Protein Folding. *Mol. Microbiol.* **78**, 203-217, (2010).

49 Cruz-Vera, L. R., Rajagopal, S., Squires, C. & Yanofsky, C. Features of Ribosome-Peptidyl-tRNA Interactions Essential for Tryptophan Induction of tna Operon Expression. *Mol. Cell* **19**, 333-343, (2005).

50 Seidelt, B. *et al.* Structural Insight into Nascent Polypeptide Chain–Mediated Translational Stalling. *Science* **326**, 1412-1415, (2009).

51 Bhushan, S. *et al.* Structural basis for translational stalling by human cytomegalovirus and fungal arginine attenuator peptide. *Mol. Cell* **40**, 138-146, (2010).

52 Nakatogawa, H. & Ito, K. The ribosomal exit tunnel functions as a discriminating gate. *Cell* **108**, 629-636, (2002).

53 Zhang, Y., Wolfle, T. & Rospert, S. Interaction of nascent chains with the ribosomal tunnel proteins Rpl4, Rpl17, and Rpl39 of Saccharomyces cerevisiae. *J. Biol. Chem.* **288**, 33697-33707, (2013).

54 Burridge, C. *et al.* Nascent chain dynamics and ribosome interactions within folded ribosome-nascent chain complexes observed by NMR spectroscopy. *Chem. Sci.* **12**, 13120-13126, (2021).

**Supplementary Tables**

**Table S1.** Average K_app_ values for nascent-chain / r-protein complexes tested in this work. Data are displayed as avg±SE.

| **Construct** | **K_app_** |
| --- | --- |
| apoHmp_1-55_-RP1 | 1.2 $\pm$ 0.85 mM  (n=2) |
| apoHmp_1-140_-RP1 | 590 $\pm$ 340 μM  (n=3) |
| apoHmp_1-189_-RP1 | 1.1 $\pm$0.78 mM  (n=4) |
| apoHmp_1-189_-RP2 | 180$\pm$ 120 μM  (n=4) |
| PIR-RP1 | 58 $\pm$41 mM  (n=2) |

**Table S2.** Name, nucleotide sequence, and calculated ΔG° of unfolding for each of the oligonucleotides used in this work to generate ribosome-bound nascent chains in an *E. coli* cell-free system. ΔG° was calculated using an online calculator (biosyn.com).

| **Construct** | **Oligonucleotide sequence** | **ΔG°unf (kCal** **• mol^-1^)** |
| --- | --- | --- |
| **Anti-ssrA** | TTAAGCTGCTAAAGCGTAGTTTTCGTCGTTTGCGACTA | **54.2** |
| **snc-tRNA** | GCGTCAAGCAT | **12.4** |
| **ApoHmp_1-32_**  **ApoHmp_108-140_**  **ApoHmp_152-184_**  **ApoHmp_230-262_**  **ApoHmp_263-295_**  **ApoHmp_371-403_**  **ApoHmp_1-55_**  **ApoHmp_1-140_**  **ApoHmp_1-189_**  **PIR_1-91_** | CATACGGTCGTAGAAATGGGCGGTTAACTTTGGCCCCG  TTAGTTATAGATTTCCGCCTCGCGATTGATAAATACAT  TATTCTGCCACTGCGCCACCGTCGACCGGC  CGGAGCGACCAGTTTCACGACATCGCCACCA  ATGGAACCAGTTCACTTGTGCTGTGTGGCCTGCTTTTG  CAGCACCTTATGCGGGCCAAAGCATTCGTAATG  AAGTGCAACTAAGCGGTAATGCGGACCAATGAG  TTAGTTATAGATTTCCGCCTCGCGATTGATAAATACAT   GAGATATTGCCCCGGACGGTATTCTGCCACTGCGCCAC  GTGGTGGTGGTGGTGGTGCTCGAGTGCGGCCGCAAGCT | **57.9**  **48.9**  **49.2**  **49.0**  **54.9**  **47.9**  **49.3**  **48.9**  **59.2**  **63.9** |

**Supplementary Figures and Legends**

**
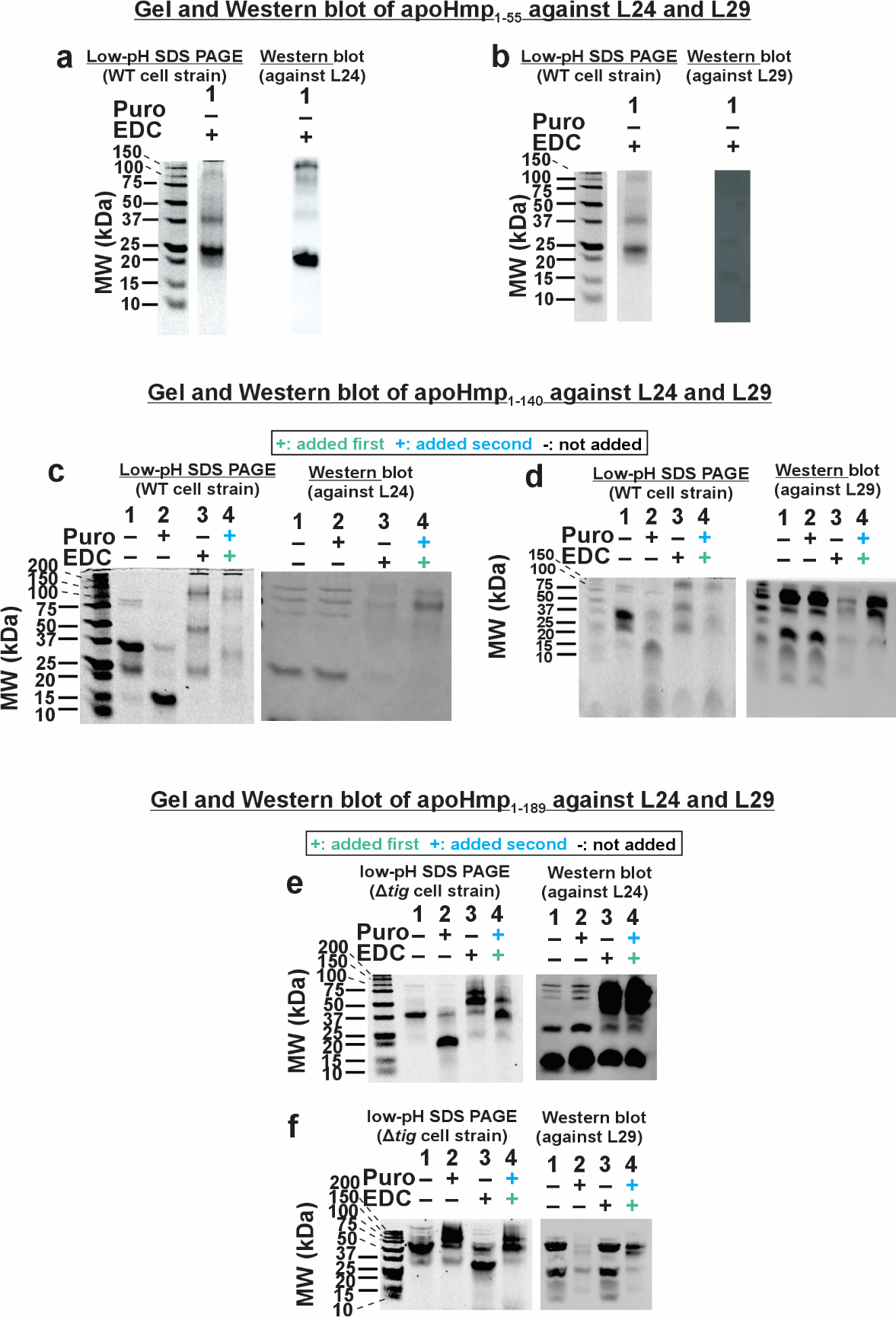
Figure S1**

**Fig. S1.** Identification of ribosomal proteins crosslinked to apoHmp_1-140_ RNCs via Western blotting. Left, low-pH 10% SDS-PAGE analysis of N-terminal fluorescently-labeled apoHmp_1-55_ RNCs generated via transcription-translation in an *E. coli* S30 cell-free system followed by Western blotting (right) employing antibodies against ribosomal proteins (a) L24 and (d) L29. Identification of ribosomal proteins crosslinked to apoHmp_1-140_ RNCs via Western blotting. Left, low-pH 10% SDS-PAGE analysis of N-terminal fluorescently-labeled apoHmp_1-140_ RNCs generated via transcription-translation in an *E. coli* *Δtig* S30 cell-free system followed by Western blotting (right) employing antibodies against ribosomal proteins (c) L24 and (d) L29. See Methods for Western blotting and antibodies details, representative data, out of n = 2, are displayed. Identification of ribosomal proteins crosslinked to apoHmp_1-189_ RNCs via Western blotting. Left, low-pH 10% SDS-PAGE analysis of N-terminal fluorescently-labeled apoHmp_1-189_ RNCs generated via transcription-translation in an *E. coli* *Δtig* S30 cell-free system followed by Western blotting (right) employing antibodies against ribosomal proteins L24 (e) and L29 (f). See Supplementary Methods for Western blotting and antibodies details. Representative data, out of n = 2, are displayed. Uncropped gel images for all panels can be found in Figure S11.

**Figure S2**

**
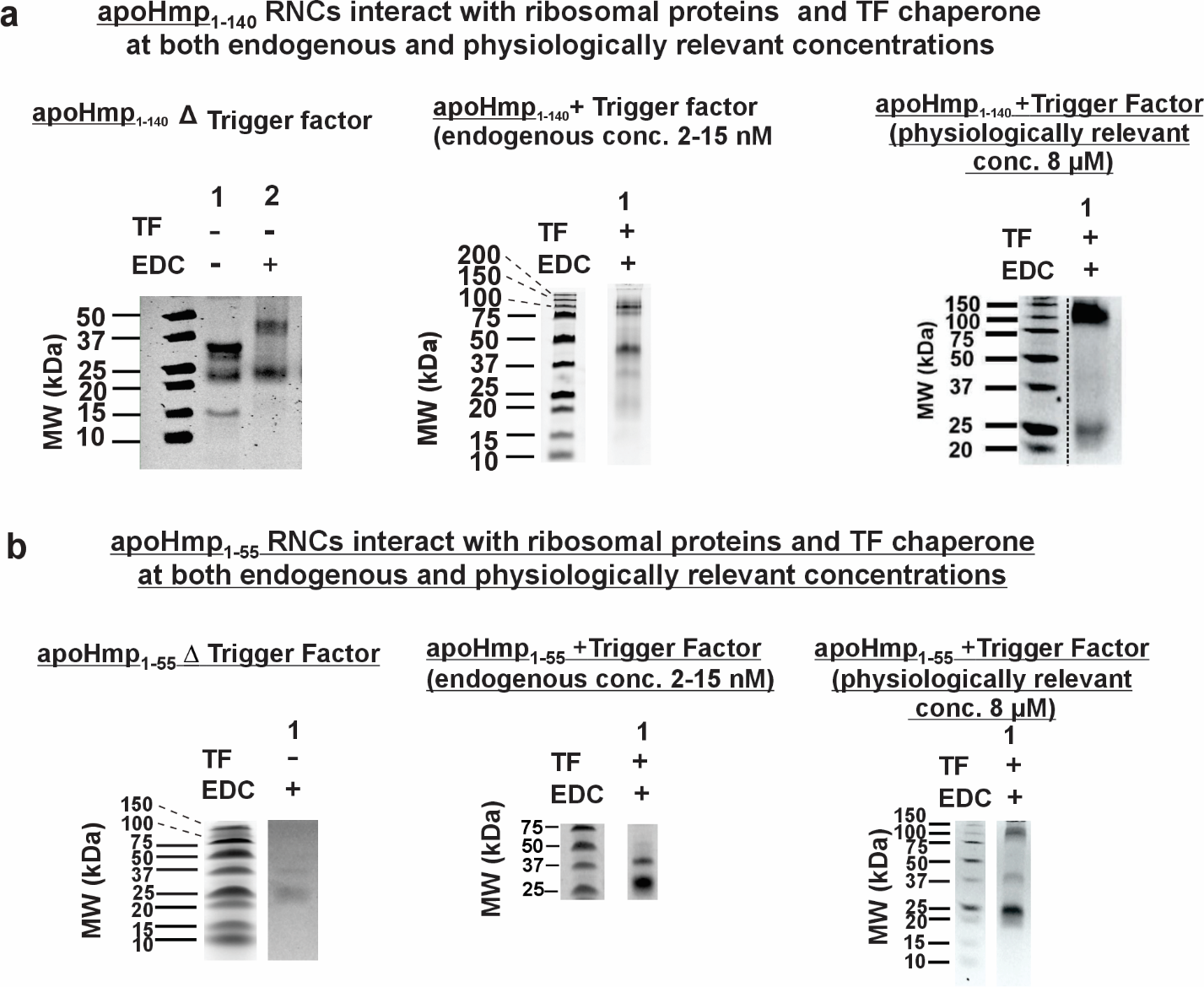
**

**Fig. S2.** (a) ApoHmp_1-140_ at various concentrations of trigger factor molecular chaperone. Concentration of trigger factor increases from left gel to right gel. (d) ApoHmp_1-55_ at various concentrations of trigger factor molecular chaperone. Concentration of trigger factor increases from left gel to right gel. See methods for more information about chaperone concentration. Uncropped gel images can be found in Figure S11.

**Figure S3**

**
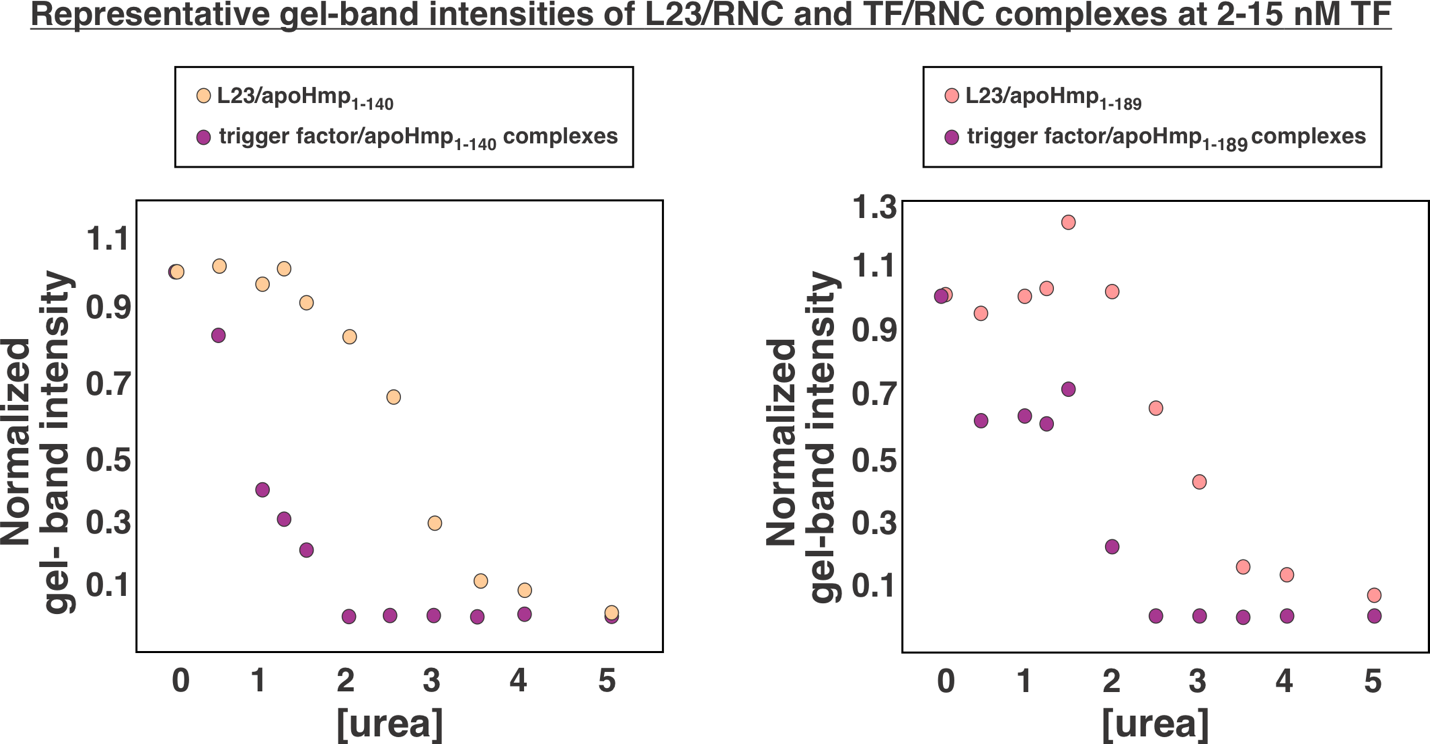
**

**Fig. S3.** Representative normalized gel-band intensities of RP1 (L23/apoHmp_1-140_ or L23/apoHmp_1-189)_ complexes and TF (TF/ apoHmp_1-140_ or TF/ apoHmp_1-189)_ complexes plotted as a function of urea concentration for (a) apoHmp_1-140_  (n=3), and (b) apoHmp_1-189_ (n=4). TF denotes the trigger factor chaperone (2-15 nM).

**Figure S4
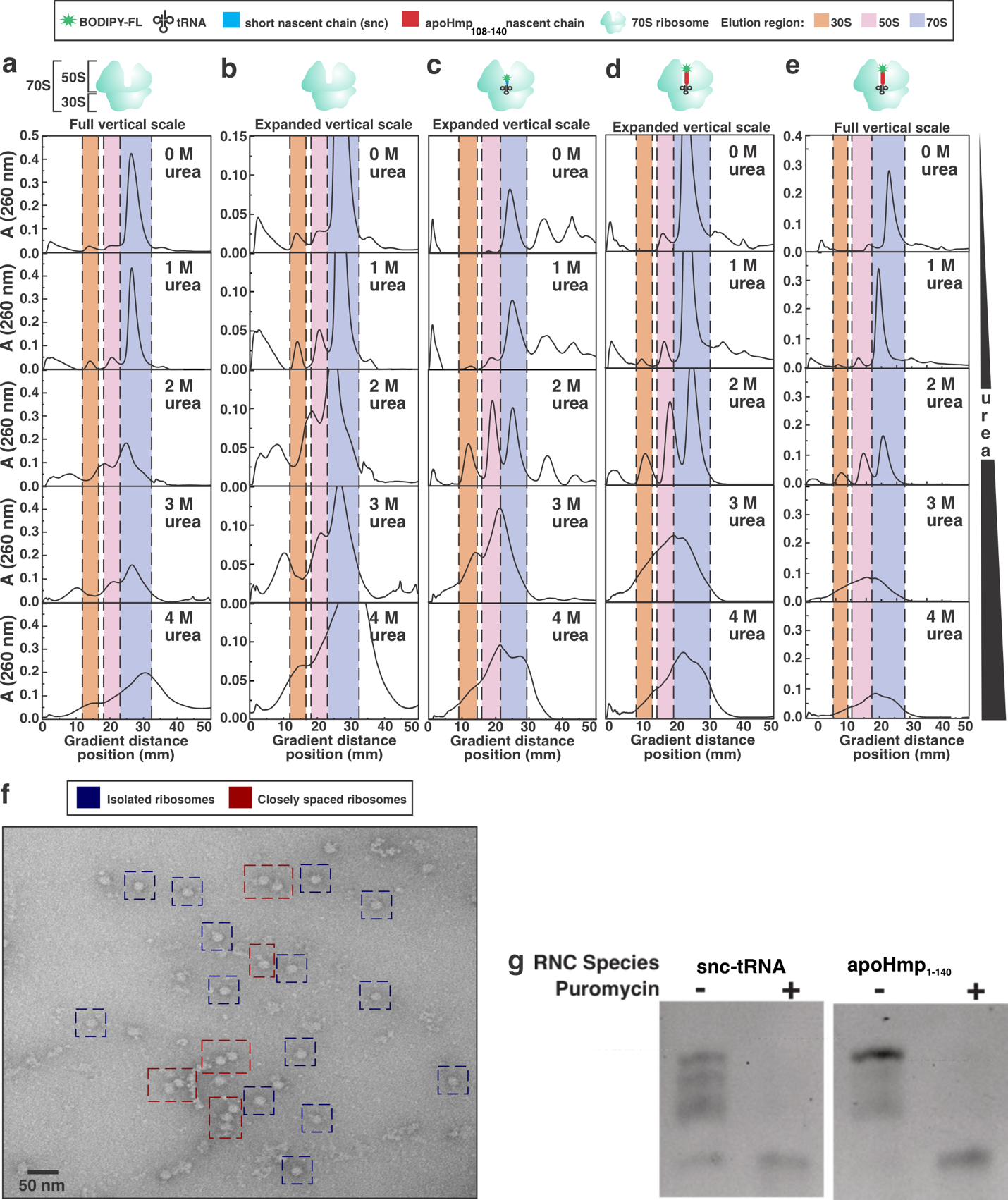
**

**Fig. S4.** Sucrose gradient profiles collected at 260nm for both empty 70S ribosomes (a), the same 70S ribosome profile is set to the same y-axis scale as the short (1-2 amino acid) nascent chains (snc-tRNA), (b) ribosomes harboring short (1-2 amino acid) nascent chains (c), ribosomes bearing a longer nascent-peptide chain (d), and the same ribosomes bearing a longer nascent-peptide chain at the full vertical scale (e) are shown for increasing concentrations of urea. Profiles were collected at 260 nm. (f) A TEM image of the snc-tRNA is shown. The dark blue squares show what are likely isolated 70S ribosomes and the red squares show closely spaced ribosomes. (g) Bound and released snc-tRNA and apoHmp_1-140_ nascent chains with the addition of puromycin (released) or without (bound) on a low pH 10% SDS-PAGE gel. Uncropped gel image can be found in Figure S12.

**Figure S5**

**
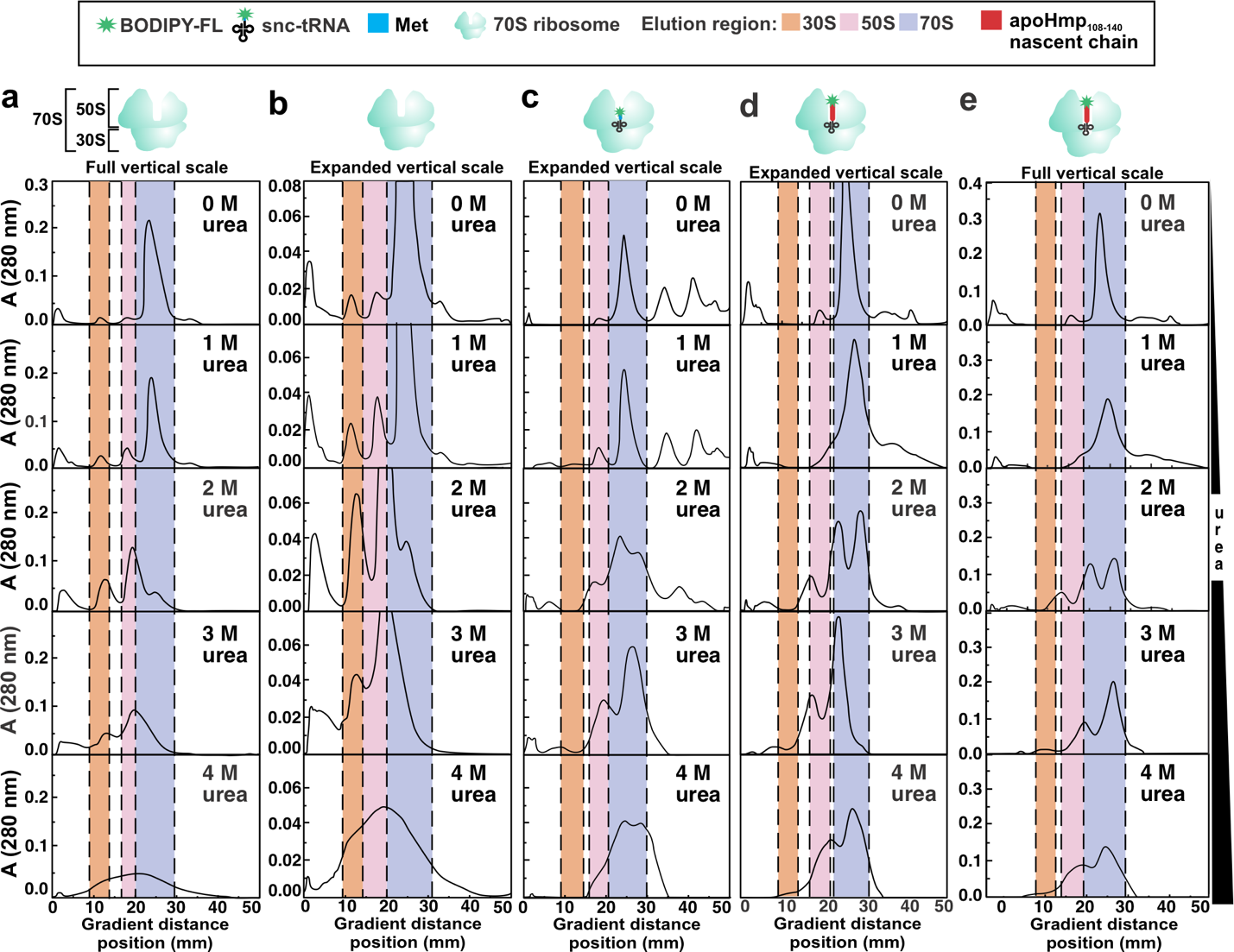
**

**Fig. S5.** Sucrose gradient profiles collected at 280nm for both empty 70S ribosomes (a), the same 70S ribosome profile is set to the same y-axis scale as the short (1-2 amino acid) nascent chains (snc-tRNA), (b) ribosomes harboring short (1-2 amino acid) nascent chains (c), ribosomes bearing a longer nascent-peptide chain (d), and the same ribosomes bearing a longer nascent-peptide chain at the full vertical scale (e) are shown for increasing concentrations of urea. Profiles were collected at 280 nm.

**Figure S6**

**
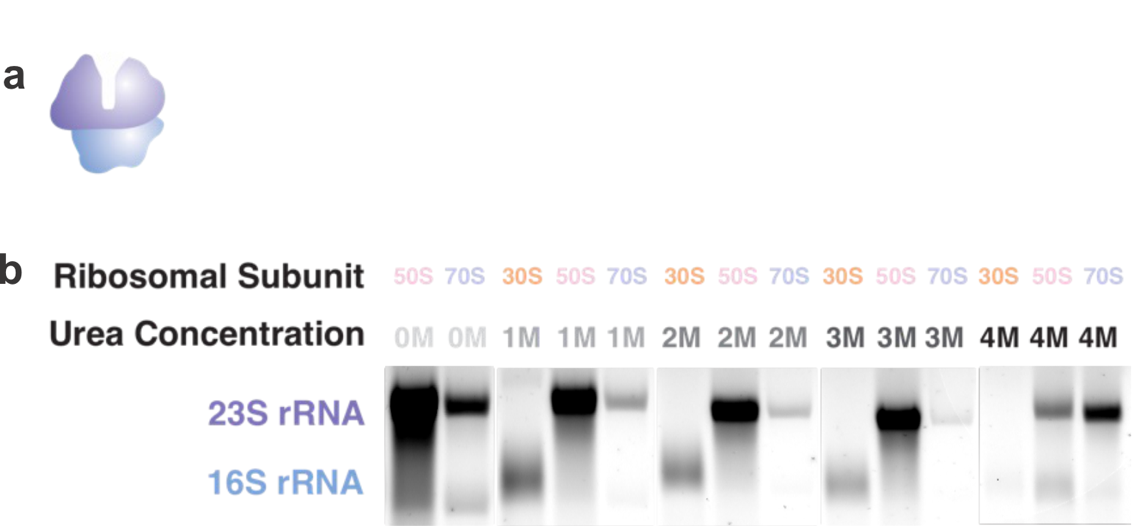
**

**Fig. S6.** (a) The 70S bacterial ribosome contains two subunits, the 30S (blue) and the 50S (purple), which contain the 16S and 23S rRNA respectively. (b) Representative agarose gel stained with ethidium bromide for the 30S, 50S and 70S of ribosome samples at increasing concentrations of urea. Subunit peak corresponds to the area in which the sample was collected. Uncropped gel images can be found in Figure S12.

**Figure S7**

**
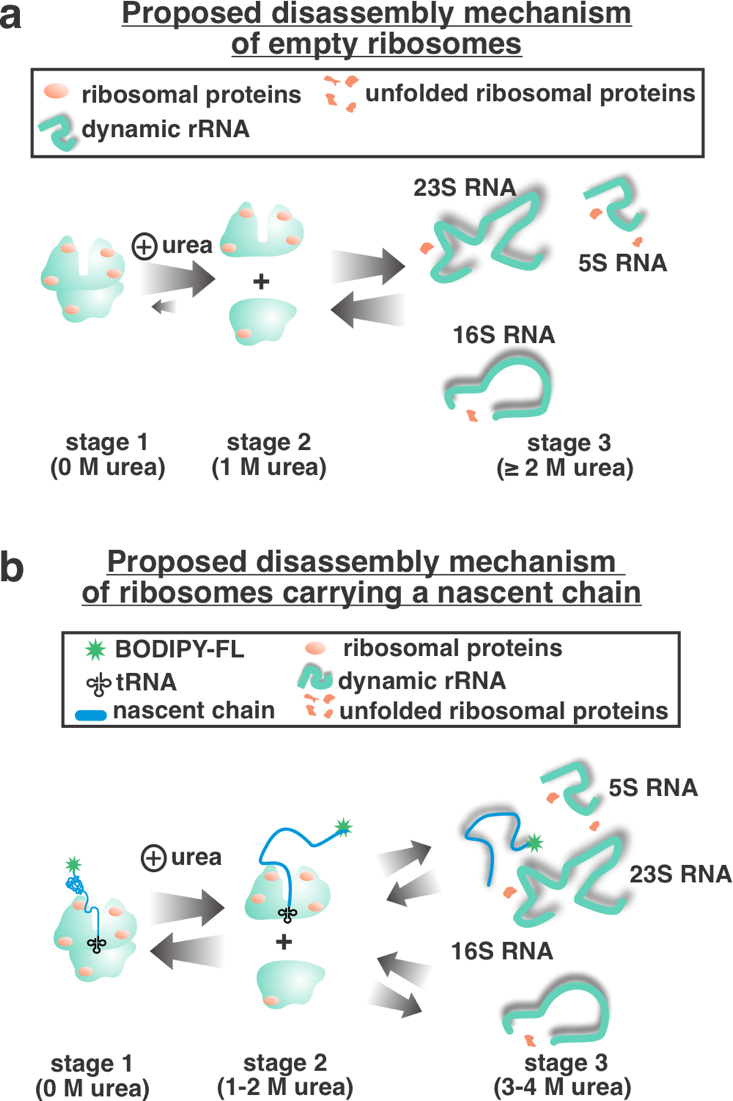
**

**Fig. S7. A proposed disassembly model for empty ribosomes and ribosome-bound nascent chains.** (a) Briefly, empty ribosomes begin to dissociate from one another at c.a. 1 M urea. At > 2 M urea empty ribosomes experience r-protein and rRNA unfolding. (b) Briefly, RNCs begin to dissociate from one another at c.a. 1-2 M urea. At > 3 M urea empty ribosomes experience r-protein and rRNA unfolding.

**
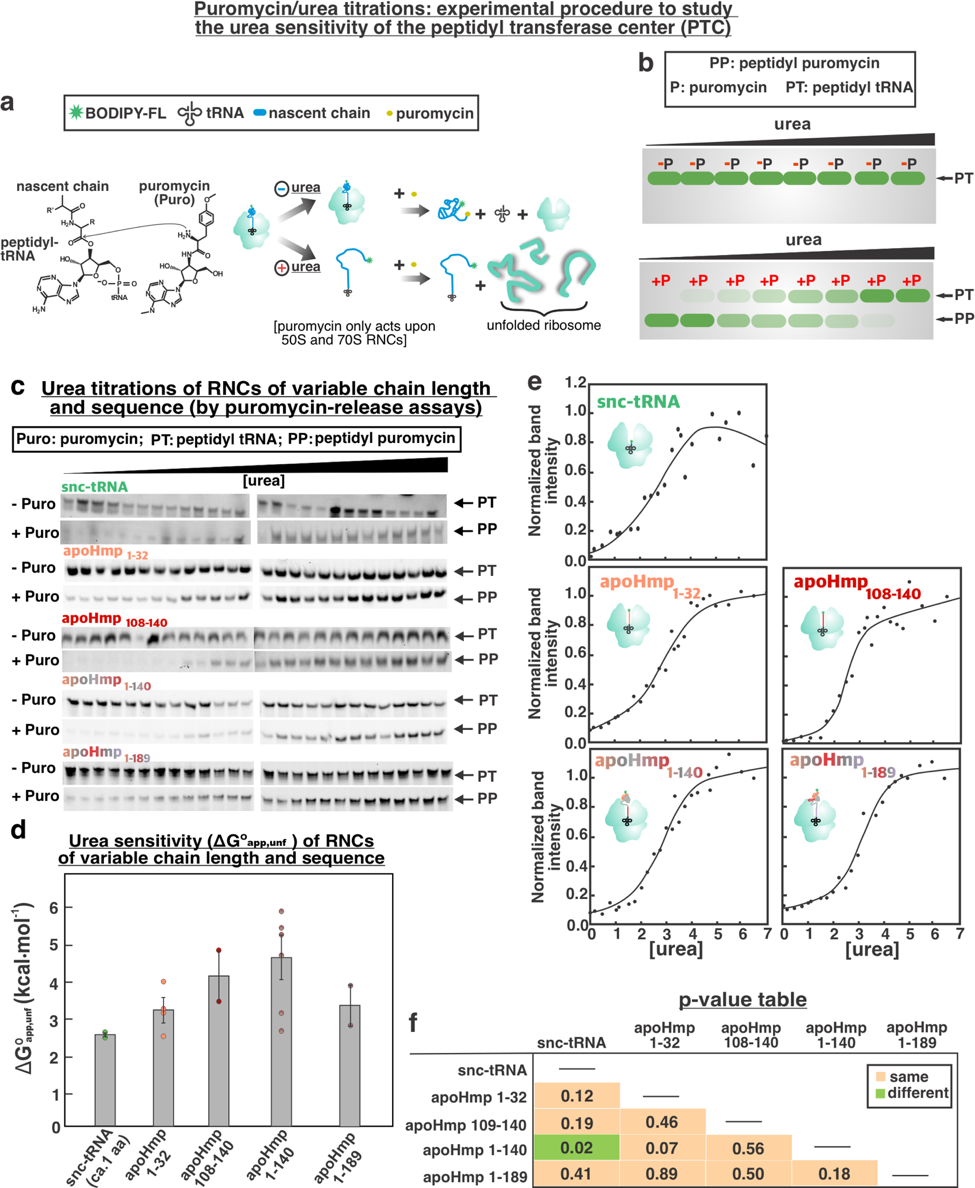
**

**Fig. S8. Sensitivity of peptidyl transferase center (PTC) of the ribosome to urea denaturation. a)** Puromycin mimics the 3’ end of the amino acyl tRNA and covalently attaches to nascent chains, promoting their release from peptidyl tRNA. This process is accompanied by a significant decrease in molecular weight. **b)** Low-pH SDS-PAGE is employed to quantify peptidyl-tRNA (PT) band intensities as a function of urea concentration. Puromycin is unable to perform its function at high urea concentration because of pervasive PTC unfolding (Supplementary Information, Fig. S1). **c)** Representative low-pH gels showing that peptidyl tRNA bands are unaffected by high urea concentrations. Addition of puromycin (1 mM) results in a decrease in peptidyl-tRNA (PT) band intensities, at low urea concentration. Uncropped gel images can be found in Figure S12. **d)** Block diagram mapping apparent unfolding free energy values (ΔG°app,unfold). Error bars denote standard errors based on 2-7 experiments. **e)**  Representative urea titration curves corresponding to the raw data in panel c. Data were fit to a two-state unfolding expression (see Materials and Methods section for details). **f)** P*-*value table for a two-tailed Student’s test assuming unequal variances (Welch’s t-test). Green and orange boxes denote statistically different and statistically equivalent data, respectively, according to a ≥ 95% confidence interval.


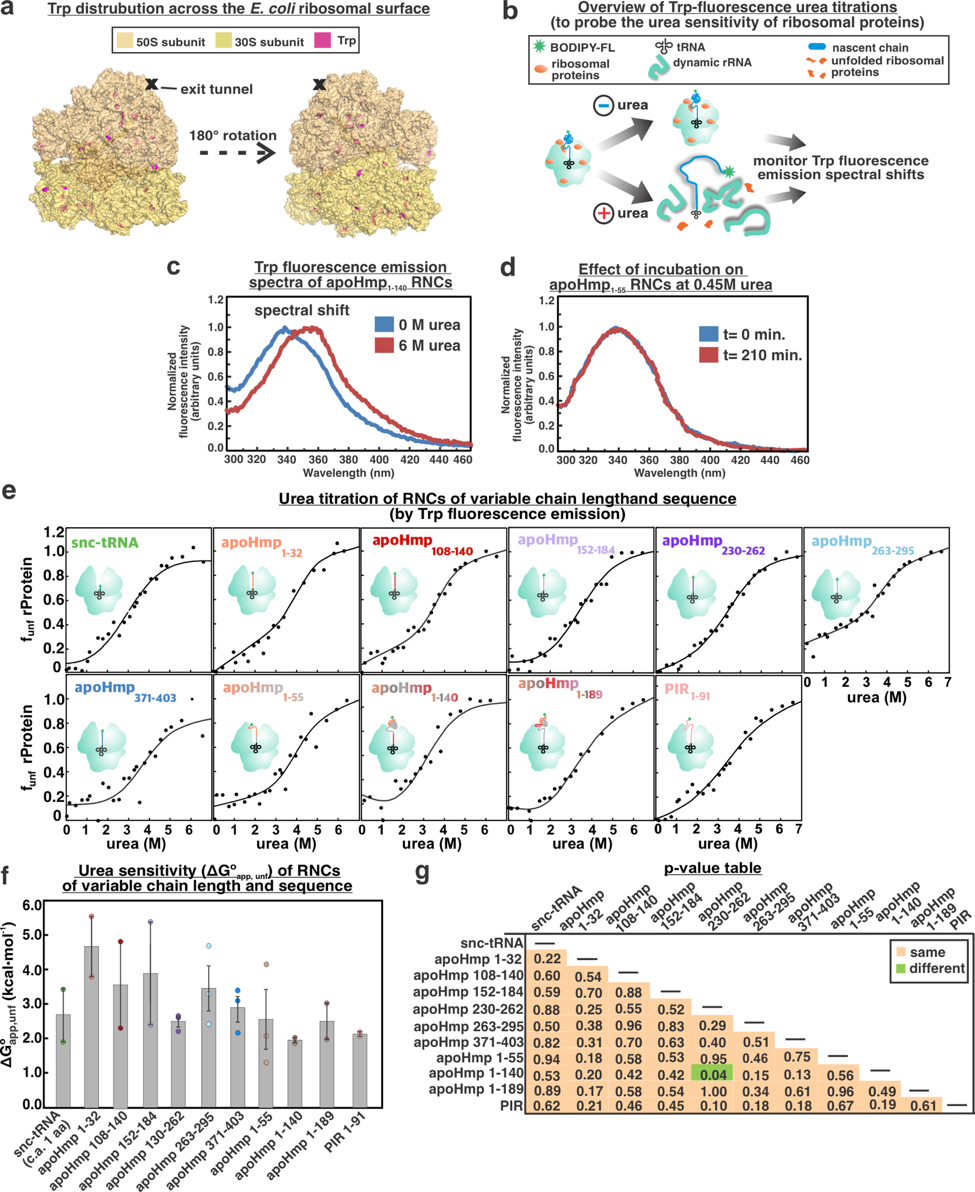


**Fig. S9. Sensitivity of ribosomal proteins to urea denaturation. a)** The r-proteins of the *E. coli* 70S ribosome include 32 tryptophans (Trp, magenta). 16 and 16 Trps are located within the 50S and 30S subunits, respectively (PDB IDs: 2WWL and 2WWQ). **b)** Scheme illustrating the methodology followed to assess r-protein stability. **c)** Trp fluorescence-emission bands become red-shifted at increasing urea concentrations due to changes towards a more polar medium. **d)** Representative urea titration curves from steady-state fluorescence of RNCs of snc-tRNA, apoHmp_1-32_, apoHmp_108-140_, apoHmp_152-184_, apoHmp_230-262_, apoHmp_263-295_, apoHmp_371-403_, apoHmp_1-55_, apoHmp_1-140_, apoHmp_1-189_ and PIR respectively. The symbol funf denotes change in the fraction of unfolded r-proteins (eq. 4). **e)** Apparent unfolding free energies (ΔG°app,unfold). Uncertainties are reported as ± SE for n=2-3. **f)** P*-*value table for the two-tailed Student’s T-test (Welch’s test) comparing ΔG°app,unfold values. Green and orange boxes denote statistically different and statistically equivalent data, respectively, according to a 95% confidence interval.

**
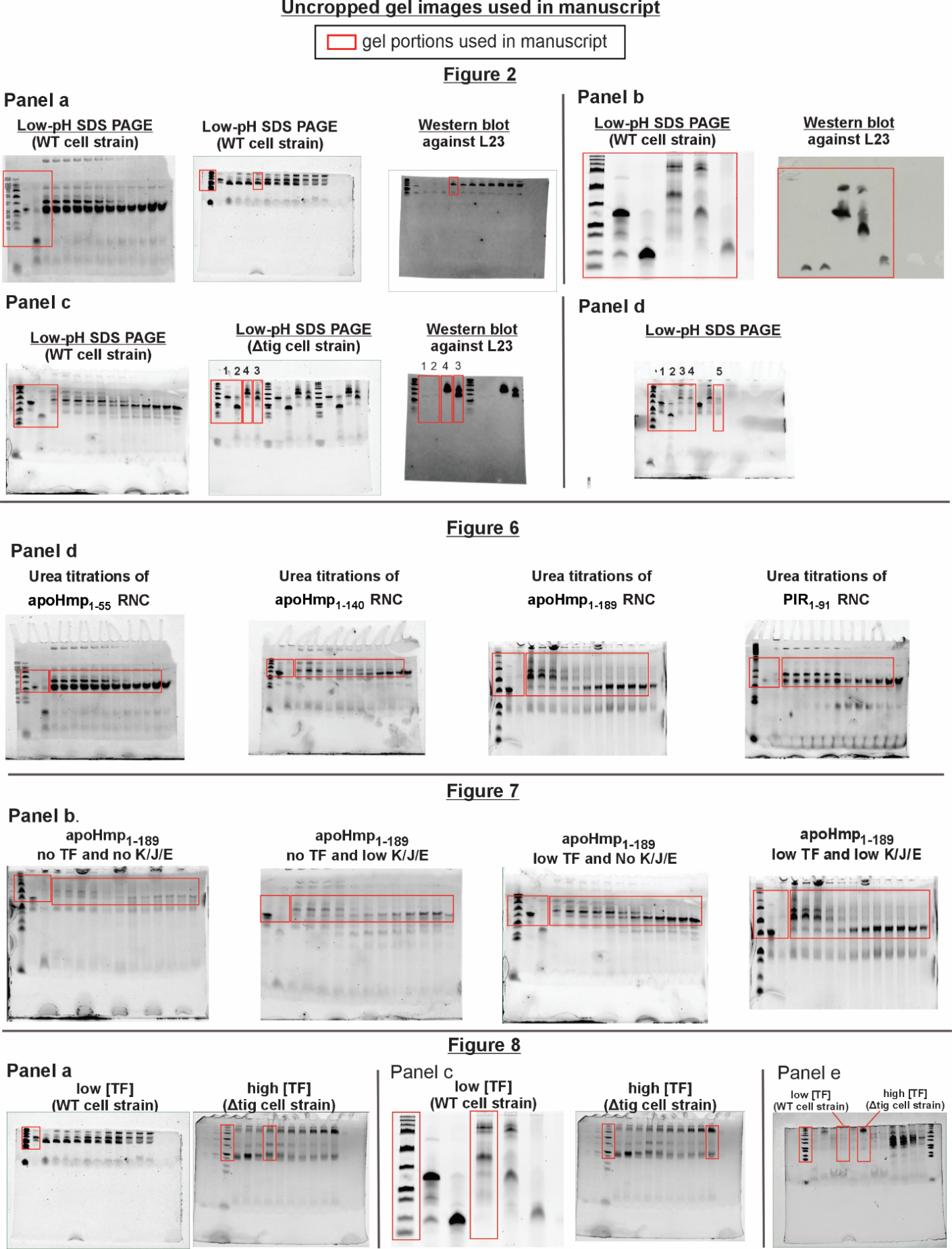
**

**Fig. S10. Original gel images.** Uncropped gel images, series I.

**
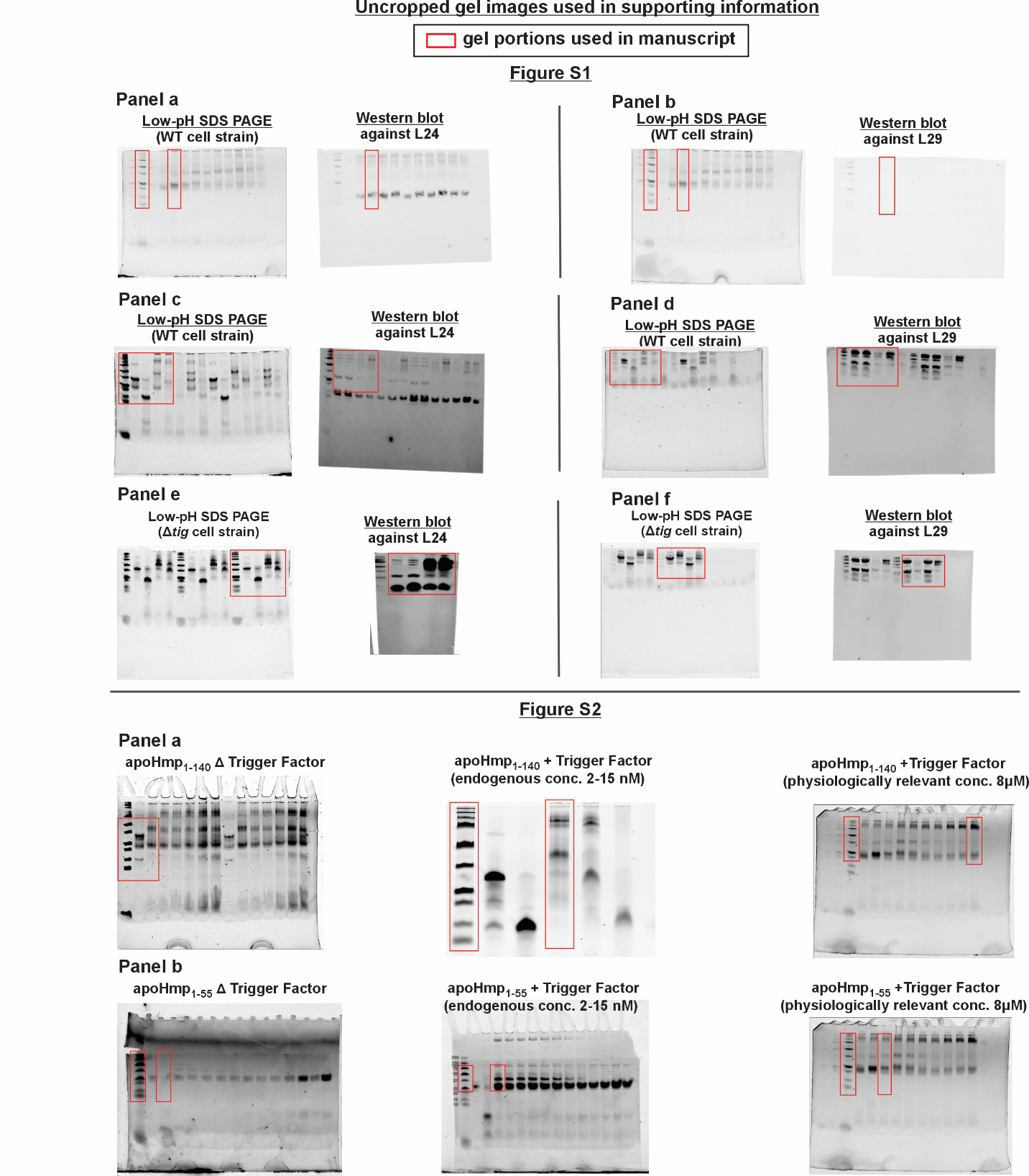
**

**Fig. S11. Original gel images.** Uncropped gel images, series II.

**
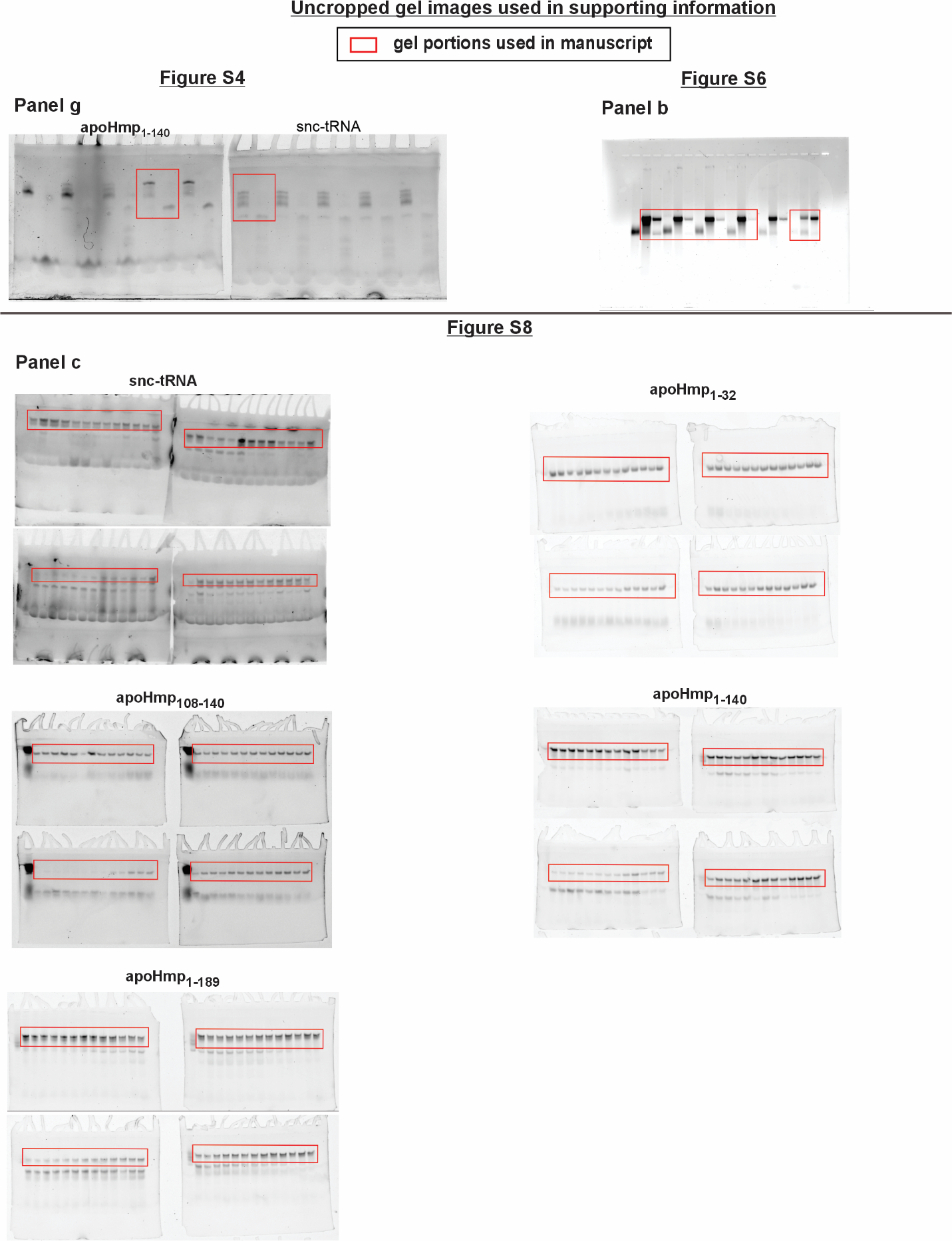
**

**Fig. S12. Original gel images.** Uncropped gel images, series III.


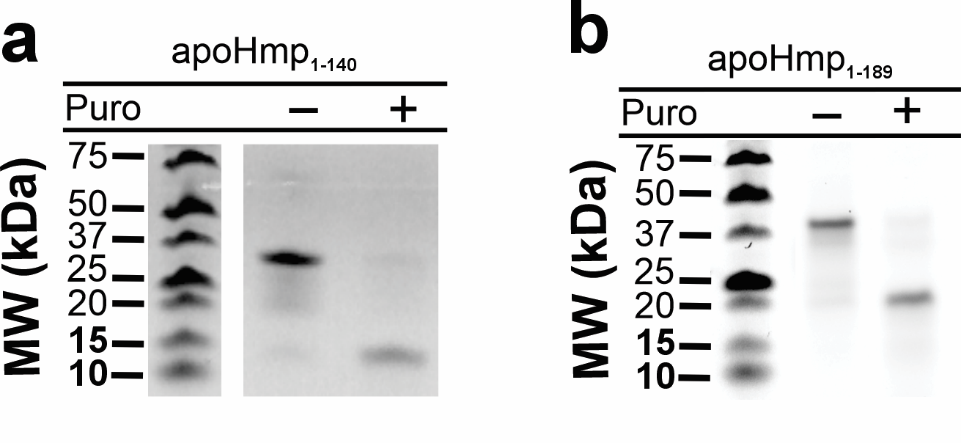


**Fig. S13. Gel images corresponding to fluorescence anisotropy-decay experiments.** This figure illustrates the low-pH SDS-PAGE analysis performed on the samples used in the fluorescence anisotropy-decay experiments of Fig. 4 (panels a and b). Specifically, gel data corresponding to **a)** apoHmp_1-140_ and **b)** apoHmp_1-189_ RNCs are shown. Low-pH SDS-PAGE analysis was performed on the same identical samples used for fluorescence, shortly after fluorescence anisotropy-decay data collection, after splitting the anisotropy sample into two equal aliquots (immediately after fluorescence data collection). Each sub-sample was flash-frozen followed by brief storage at -80 ºC). The 1^st^ sub-sample was analyzed as is, in the absence of puromycin (- Puro), and the 2^nd^ sub-sample was examined after incubation in the presence of the puromycin antibiotic (+ Puro, see Materials and Methods). The puromycin treatment was carried out to trigger RNC release from the ribosome and thereby ensure that the large majority of the sample indeed corresponds to an RNC. It is well-known that puromycin only acts upon ribosome-associated aminoacyl or peptidyl tRNAs. In addition, the low-pH SDS-PAGE analysis of the gel samples before addition of puromycin also serves the purpose of ensuring the absence of RNC chain-length heterogeneity. The above analysis was performed on each of the samples used in the entire set of fluorescence anisotropy-decay experiments. Only samples that displayed a large preponderance of ribosome-bound nascent chain nature (i.e., samples showing a predominant gel band of the appropriate molecular weight of ca. 26-28 kDa for tRNA plus the MW of the appropriate nascent chain) in the above gel assays were employed for the fluorescence anisotropy-decay data employed in this work. The above post-fluorescence gel-verification procedure has been routinely employed for all anisotropy-decay data collection that has been published by the Cavagnero group, to date ^1-6^. Uncropped gel images can be found in Figure S14.


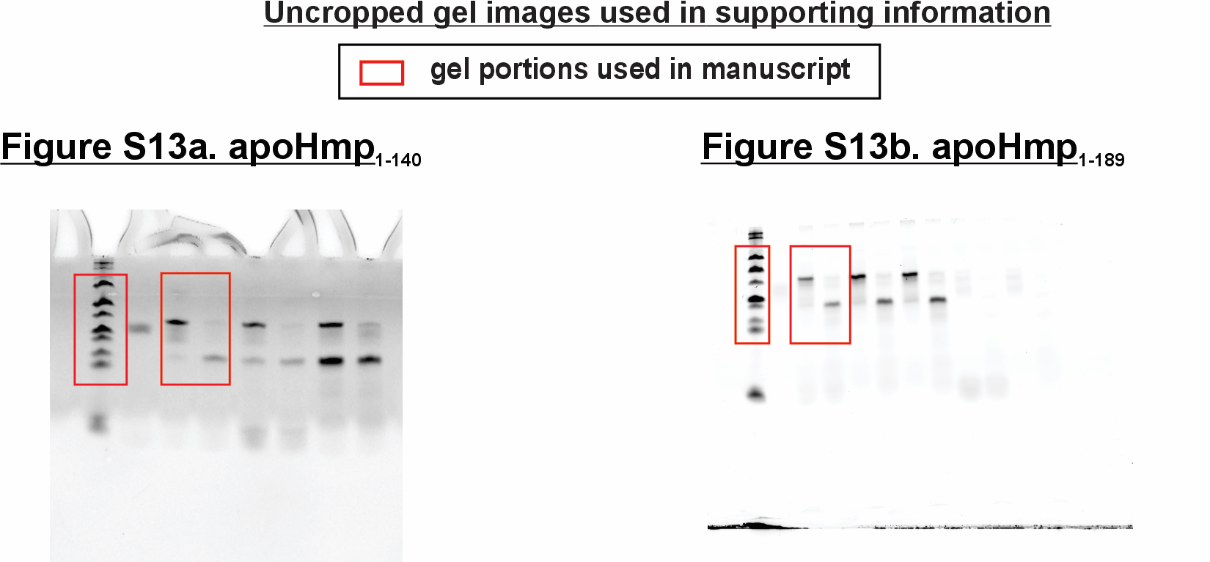


**Fig. S14. Original gel images.** Uncropped gel images, series IV.
